# Supplementary material for: EU-ETS emergency reserve price curbs coal use and shields consumers during natural gas price shocks
Source: Nat Commun. 2026 May 25;17:4637. doi: 10.1038/s41467-026-73559-2 (PMC13199404; doi:10.1038/s41467-026-73559-2)
Supplement: Supplementary file 1 — Supplementary Information [file 41467_2026_73559_MOESM1_ESM.pdf]

Supporting Information for "EU-ETS emergency reserve price curbs coal use and shields consumers during natural gas price shocks"

April 30, 2026

Contents

1 Supplementary Discussion **2**

1.1 Electricity and Commodity markets 2

1.2 Dataset and sample 4

1.3 Robustness Checks 5

1.3.1 Econometric Model of Coal Responsiveness 5

1.3.2 Econometric Model of Pass-Through 33

1.3.3 Relative Responsiveness Index 35

1.3.4 Policy analysis 38

2 Supplementary Tables **45**

3 Supplementary Figures **47**

1 Supplementary Discussion

1.1 Electricity and Commodity markets

In this supplementary discussion we provide additional information on natural gas, coal, and wholesale electricity markets in Europe.

Natural Gas Markets Prior to the nineties, natural gas markets across Europe were heavily regulated by government-owned and controlled firms. The next decade resulted in a series of market liberalizations and deregulations, culminating in the European Union directive in 1998 (98/30/EC), eventually creating multiple trading hubs across the nation where market participants act to set a price and delivery dates for natural gas. Several legislative actions further strengthened competition across the EU, bolstering the requirements meant to disentangle the supply from distribution of natural gas [1, 2].

Since the early 2010s, the Dutch Title Transfer Facility (TTF) hub has grown substantially to become the leading hub in Europe (surpassing the British National Balancing Point) and become one of the global pricing benchmarks for natural gas alongside the Japan Korea Marker (JKM) and US Henry Hub [2]. Due to its proximity to storage facilities and important port terminals, it proved to be the most connective to other European ports and markets. The TTF is priced in EUR/MWh, which traders prefer to pence/therm that is done in NBP contracts [3].

The physical infrastructure between and within countries determines the level of natural gas connectivity and the ease of arbitrage between prices at different locations. While natural gas pipelines were the main methods of transportation, re-gasification terminals have been increasing in number due to the rising popularity of LNG. Price differences at all European Hubs with sufficient trading volume are minimal and usually the cost of transportation; even more so, these differences are decreasing due to the development of swap contracts between gas shipping traders. As such, these hubs are strongly correlated and increasing convergent [1, 4–8]. Most European natural gas has historically been sourced from Russia and Norway via physical pipelines. Following the onset of the war, from summer 2022 on (i.e. outside of our estimation sample period), Norway and other suppliers such as Algeria and the United Kingdom have increased their supply flow to European countries, while countries such as the US and Qatar have exponentially increased their sales of liquid natural gas to make up for this lack of supply.

The pricing of natural gas is thus determined on these international hub markets that are often results of not only demand and supply forces, but also market speculation of traders. As such, geopolitical events in that may rattle markets or actors that supply natural gas can greatly impact the price. While actions of a significant market seller of natural gas can evidently affect the price of natural gas (i.e., Russian army buildup and invasion of Ukraine), it is also true that any geopolitical

tension in proximity to these actors can also induce volatile and price spikes (i.e., sociopolitical instability in Israel and Gaza). Importantly, these massive price fluctuations are often due to exogenous factors, unrelated to the workings of the natural gas market. Most relevant to the period of our paper, Russia disrupted EU gas supplies starting in Q3 2021 through Gazprom not refilling gas storage sites and randomly interrupting deliveries via pipelines such as Nordstream. Our empirical strategy exploits these geopolitical events that induce exogenous variation in gas prices to estimate key response mechanisms for policy analysis.

In particular, we use the TTF month-end futures prices, which are most used by gas-fired generators to hedge the spark spread risk [9]. These prices are thus the ones most observed by market participants and used to determine the profit margin earned by buying natural gas and producing electricity [10, 11]. Furthermore, futures prices are shown to be more reactive to market information than spot prices, since the futures market is easily accessible to financial traders, without interest in physical delivery [12–14]. As such, short-term (month) futures provide the most valid price signals, while spot prices are often impacted by unpredictable short-run conditions against which generators are hedged via future trading [14].

Coal markets Since before the inception of the European Union, coal has been at the forefront of European markets. The European Coal and Steel Community (pre-cursor of the European Union) set the foundations of European integration and economic cooperation. Today, the most liquid and utilized coal hub is the Amsterdam Rotterdam Antwerp (ARA) hub, imported through Northwestern Europe. This is the largest derivative market for coal, referring to steam coal that takes into consideration the international transportation costs (CIF, Cost Insurance and Freight) [15]. The prices set at this hub are used as a benchmark for European generators, and thus these prices reflect the fuel cost utilized in electricity production under hard coal. The units for this price are in USD/metric tonne, and thus we convert to EUR/MWh based on an assumed heat rate and conversion factor of tonnes to MWh of 8.14. Similarly to the TTF hub gas prices, month-end futures are the most liquid contract on the market. In contrast to the natural gas markets, coal markets are much less volatile. Lignite is not traded on these markets as much of it is sourced in place, but generally follows the coal benchmark prices.

Though to a lesser extent than natural gas markets, European coal prices are affected by international geopolitical conditions, such as the Russian invasion of Ukraine, or even supply constraints in major producers and exporters, such as Australia or Indonesia.

Electricity markets Prior to liberalization reforms in European markets, each country's power sector operated according to its own rules under heavy regulation consisting of vertically-integrated utilities responsible for generation, transmission, distribution, and retail of electricity. European

Union directives prompted this liberalization by creating competitive wholesale markets with numerous private generators in lieu of vertically integrated monopolies [16, 17]. Furthermore, it created common rules for countries to allow for integration towards a single power market. While the current state of integration is far from perfect, the foundations in place allow for the partial interconnection of electricity across borders.

Most importantly, the common framework led to the creation of the day-ahead and intraday electricity markets [18]. The day-ahead market consists of generators bidding for electricity at a specific hour of the day of the following day, under different demand forecasts, while the intraday market allows for balancing bids to the deviations in supply and demand from the day-ahead forecasts. While the intra-day market is essential to match supply and demand at all times of the day, the day-ahead market primarily acts to set the wholesale electricity price as it is directly impacted by the price of fuels used by generators that participate, which is why it remains the focal point of most paper analyses (including this one).

Every noon, an auction is held to determine the quantities and prices of electricity supplied for the next 24 hours the following day. Generators bid the price in EUR/MWh at which they are willing to supply a quantity of electricity at a given time, determined by its operational cost (mainly consisting of fuel costs and emissions costs). The market operator matches each generators' bids to the supply of electricity, creating the merit order, which essentially reflects the ascending order of costs of fuels of the different generators. Thus, the lower-bidding (and hence lower marginal cost) generators are dispatched first (e.g., intermittent renewables, nuclear, etc.). The intersection of the supply curve of generators with the forecasted demand sets the wholesale electricity market-clearing price for each hour of the day. The last, and most expensive, generator dispatched at each hour is termed the "marginal generator," as it sets the market price due to the uniform-price auction. Thus, all generators, including those with much less marginal costs, are paid the exact same price for electricity supply. Given that the fuel cost is often the prime determinant of bids, the wholesale market price of electricity is highly dependent on these commodity prices, such as natural gas. Thus, the composition of the electricity mix is another determinant of a market's level of pass-through of natural gas prices to electricity prices.

1.2 Dataset and sample

The data on hourly wholesale electricity prices, load, and generation were collected via API from ENTSO-E, which covers European Transmission System Operators (TSO) across 35 European countries. A unique API key was obtained to download these data for 14 EU countries using the EntsoePandas Python client and appropriate zone codes (EIC) outlined on their website [1] Electric-

[1] https://transparency.entsoe.eu/content/static_content/Static%20content/web%20api/Guide.

ity generation sources used include natural gas, hard coal, lignite coal, nuclear, solar, wind, and hydro-run-of-river. As mentioned in the Methods, Irish electricity prices could not be obtained and thus Ireland is only analyzed during the first part of our paper (Fig. 2). Settlement prices of futures for natural gas, coal, and carbon allowances in the EU ETS were obtained from Thomson Reuters Eikon. For days in which these prices are unavailable due to market closures, the previous day closing value is used, which reflects the appropriate market opportunity cost of using the fuel that the point in time. All commodities are converted to EUR/MWh for calculations (see Methods).

Our sample period for this study was chosen as April 1 2021 to May 31 2022, as it best reflects the onset of the natural gas price shock from the Russian army buildup of preparation of the Ukraine invasion, up to three months after the invasion in which governments started enacting policies that would confound commodity and wholesale markets. For one, the REPowerEU package, meant as an EU-wide comprehensive response to the crisis, was proposed in May 2022. Proposals such as the natural gas price cap, were meant to be implemented starting early 2023. While various initiatives were proposed earlier by national governments, they are primarily targeted towards shielding and relieving consumer retail bills, as opposed to wholesale markets. Almost all countries provided some form of partial retail price compensation to companies and households, as well as VAT reduction, starting December 2021, but sought not influence the wholesale market. Specifically, many countries targeted vulnerable households for subsidies. It was not until the summer of 2022 that talk of capping prices in the wholesale market was pronounced (with some countries eventually adopting windfall taxes on energy producers or wholesale electricity price caps). A key example is the so-called "Iberian exception", which came into place during the summer of 2022 and essentially capped natural gas, and thus wholesale electricity prices.[2] Thus, during this period we can plausibly justify the natural gas price spikes to be exogenous. For robustness checks of different sample periods see Supp. Tab 15.

1.3 Robustness Checks

1.3.1 Econometric Model of Coal Responsiveness

We conduct a series of robustness checks to verify the validity of our results. In terms of our main regression for the responsiveness of coal generation to the relative gas price, Supp. Tab. 1-13 illustrate the marginal effect estimates with different specifications for each country, such as the addition of monthly fixed effects, day-of-week and hourly fixed effects, the inclusion of covariates such as load, intermittent renewables, and nuclear energy generation, as well as the clustering of standard errors, to arrive at our preferred specification in column (5). Importantly, while the

[2]For a comprehensive list of policies enacted by countries see: https://www.bruegel.org/dataset/national-policies-shield-consumers-rising-energy-prices

inclusion of these control variables has a noticeable (though qualitatively similar) impact on the magnitude of the coefficient in most countries, the inclusion of monthly fixed effects (column 4) is shown to have the largest impact on the estimates for most countries, with the largest differences in the Netherlands (Supp. Tab. 11), Ireland (Supp. Tab 9), and Croatia (Sup. Tab. 2) . Since a threat to our estimation strategy could be unobservable variables that are potentially correlated with both coal generation and commodity prices and trending over time, month fixed effects restrict our identifying variation to be strictly within a month. For example, we statistically allow comparisons between prices on June 7 and June 20, but not between September 5 and August 6. [3] Clustered errors yields qualitatively similar results as robust standard errors, with the exception of Croatia and Romania (Supp. Tab. 12). Furthermore, using Newey standard errors yields qualitatively similar results to our main specification of clustering standard errors by day, which is the unit of variation of the treatment variable (natural gas and coal prices). To validate our choice of standard errors clustering for heteroskedasticity and autocorrelation concerns, the ACF of the regression residuals for each country confirm strong intraday correlation, and the PACF show dependence of the first few lags, in line with operational fuel-switching dynamics and inertia or ramping of coal generation (Supp. Tab. 22-23). An additional check including a lagged regressor of the relative price of the previous day (24 hours before) is included, yielding virtually identical results (Supp. Tab. 20). .

We further conduct robustness checks for the sample period analyzed with the main estimates for each subperiod shown in Supp. Tab. 15. Specifically, we shorten the time period in four different ways (Supp. Tab 16-19). While our results remain qualitatively similar across all subperiods, certain countries (Czechia, Denmark, Spain, Finland) exhibit a significant responsiveness effect only during the entire sample period, which may indicate that in these countries either certain thresholds of the relative price need to be reached in order to see a substitution effect, or that these thresholds were already reached and there is no significant substitution change observed during the smaller subsamples. On the other side, Hungary is shown to have significant responsiveness effect for all smaller subsamples but not the full sample, which may indicate idiosyncrasies or confounders in the Hungarian market that obstruct us from observing the desired estimate. Nevertheless, our full sample period reflects a plethora of relative price variation that allows us to uncover the substitutability of these countries, with most countries showing qualitatively similar patterns. Lastly, we assess a robustness check with different functional forms using our main specification

[3] An exception to the month fixed effects approach is Greece, where (in contrast to the rest of Europe) a smoothing price mechanism based on the average price of the previous month's TTF price is applied (see https://www.rae.gr/anakoinoseis/31535/ and https://www.kathimerini.gr/economy/562016074/sta-ypsi-oi-times-toy-fysikoy-aerioy-gia-olo-to-2023/). As such, month fixed effects would absorb all the meaningful variation in natural gas prices, and so we instead use a linear time trend to control for any spurious unobserved trends. This is also done for the econometric model of natural gas price pass-through.

and sample period (Supp. Tab. 14). While for the vast majority of countries the BIC is minimized with our main specification compared to the less complex ones omitting the squared or linear term, few countries exhibit similar BICs with the quadratic specification (Romania, Hungary, Croatia). A few countries also have a minimum BIC under the levels specification, where the dependent variable coal generation is not log-transformed. However, to account for the large variation in coal capacity of different countries we use a log-transform for interpretation purposes. An additional robustness check is concluded by running the pooled regression to get an average estimates for all countries, including country fixed effects and thus controlling for any EU-wide shocks. This elasticity estimate (0.21) is significant and similar to the average of the country specific estimates (0.26).

Bulgaria						
	(1)	(2)	(3)	(4)	**(5)**	(6)
VARIABLES						
Relative Price	3.71***	3.38***	3.49***	1.96***	**1.96*** **	1.96***
	(0.081)	(0.070)	(0.063)	(0.055)	**(0.244)**	(0.077)
Relative Price Squared	-1.22***	-1.16***	-1.19***	-0.69***	**-0.69*** **	-0.69***
	(0.031)	(0.027)	(0.025)	(0.021)	**(0.094)**	(0.030)
Relative Price Cubed	0.13***	0.12***	0.13***	0.08***	**0.08*** **	0.08***
	(0.004)	(0.003)	(0.003)	(0.003)	**(0.012)**	(0.004)
Intermittent Renewables (GWh)		-0.13***	-0.18***	-0.15***	**-0.15*** **	-0.15***
		(0.007)	(0.006)	(0.006)	**(0.019)**	(0.009)
Nuclear (GWh)			-0.00***			
			(0.000)			
Load (GWh)		0.37***	0.45***	0.31***	**0.31*** **	0.31***
		(0.022)	(0.017)	(0.015)	**(0.045)**	(0.020)
Load Squared (GWh)			-0.03***	-0.02***	**-0.02*** **	-0.02***
			(0.002)	(0.001)	**(0.004)**	(0.002)
Observations	10,223	10,223	10,223	10,223	**10,223**	10,223
Adjusted R-squared	0.549	0.650	0.749	0.895	**0.895**	
Month FE	NO	NO	YES	YES	**YES**	YES
Hour FE	NO	NO	NO	YES	**YES**	YES
Day-of-Week FE	NO	NO	NO	YES	**YES**	YES
SE	Robust	Robust	Robust	Robust	**Day Clustered**	Newey
Marginal Effect	0.618	0.481	0.504	0.259	**0.259**	0.259
P-value	1.00e-08	1.00e-08	1.00e-08	1.00e-08	**1.00e-08**	1.00e-08

Robust standard errors in parentheses
*** p<0.01, ** p<0.05, * p<0.1

Supplementary Tab. 1: Coal responsiveness regression estimates for Bulgaria under alternative model specifications, with column (5) being our main preferred specification. The dependent variable is log-transformed coal generation The marginal effect, shown on the bottom, is our main estimate of interest, derived through the delta method from the Relative Price Coefficients, with its p-value listed below it. The statistical test used is a two-sided t-test. * indicates significance at the 10% level, ** indicates significance at the 5% level, and *** indicates significance at the 1% level.

	Croatia					
	(1)	(2)	(3)	(4)	**(5)**	(6)
VARIABLES						
Relative Price	0.58***	0.49***	0.49***	0.45***	**0.45**	0.45***
	(0.071)	(0.072)	(0.072)	(0.134)	**(0.401)**	(0.173)
Relative Price Squared	-0.23***	-0.19***	-0.19***	-0.13***	**-0.13**	-0.13**
	(0.027)	(0.028)	(0.028)	(0.047)	**(0.141)**	(0.061)
Relative Price Cubed	0.02***	0.02***	0.02***	0.01*	**0.01**	0.01
	(0.003)	(0.003)	(0.003)	(0.005)	**(0.015)**	(0.007)
Intermittent Renewables (GWh)		-0.07***	-0.07***	-0.07***	**-0.07*** **	-0.07***
		(0.011)	(0.011)	(0.010)	**(0.023)**	(0.013)
Load (GWh)		0.08**	0.08**	0.25***	**0.25** **	0.25***
		(0.039)	(0.039)	(0.055)	**(0.118)**	(0.072)
Load Squared (GWh)			-0.01	-0.04***	**-0.04**	-0.04**
			(0.009)	(0.012)	**(0.023)**	(0.015)
Observations	7,981	7,981	7,981	7,981	**7,981**	7,981
Adjusted R-squared	0.085	0.096	0.096	0.205	**0.205**	
Month FE	NO	NO	YES	YES	**YES**	YES
Hour FE	NO	NO	NO	YES	**YES**	YES
Day-of-Week FE	NO	NO	NO	YES	**YES**	YES
SE	Robust	Robust	Robust	Robust	**Day Clustered**	Newey
Marginal Effect	-0.0277	-0.0267	-0.0267	0.0836	**0.0836**	0.0836
P-value	1.00e-08	2.17e-08	2.17e-08	3.51e-08	**0.0558**	1.92e-05

Robust standard errors in parentheses
*** p<0.01, ** p<0.05, * p<0.1

Supplementary Tab. 2: Coal responsiveness regression estimates for Croatia under alternative model specifications, with column (5) being our main preferred specification. The dependent variable is log-transformed coal generation The marginal effect, shown on the bottom, is our main estimate of interest, derived through the delta method from the Relative Price Coefficients, with its p-value listed below it. The statistical test used is a two-sided t-test. * indicates significance at the 10% level, ** indicates significance at the 5% level, and *** indicates significance at the 1% level.

Czech Republic						
	(1)	(2)	(3)	(4)	**(5)**	(6)
VARIABLES						
Relative Price	1.53***	1.82***	1.94***	1.28***	**1.28*** **	1.28***
	(0.063)	(0.053)	(0.055)	(0.062)	**(0.280)**	(0.087)
Relative Price Squared	-0.37***	-0.56***	-0.59***	-0.43***	**-0.43*** **	-0.43***
	(0.024)	(0.021)	(0.021)	(0.025)	**(0.111)**	(0.035)
Relative Price Cubed	0.03***	0.06***	0.06***	0.05***	**0.05*** **	0.05***
	(0.003)	(0.003)	(0.003)	(0.003)	**(0.013)**	(0.004)
Intermittent Renewables (GWh)		-0.15***	-0.15***	-0.05***	**-0.05*** **	-0.05***
		(0.005)	(0.005)	(0.007)	**(0.017)**	(0.009)
Nuclear (GWh)			-0.00***			
			(0.000)			
Load (GWh)		0.14***	0.15***	0.12***	**0.12*** **	0.12***
		(0.014)	(0.014)	(0.015)	**(0.044)**	(0.021)
Load Squared (GWh)			-0.00***	-0.00	**-0.00**	-0.00
			(0.001)	(0.001)	**(0.002)**	(0.001)
Observations	10,224	10,224	10,224	10,224	**10,224**	10,224
Adjusted R-squared	0.548	0.702	0.703	0.781	**0.781**	
Month FE	NO	NO	YES	YES	**YES**	YES
Hour FE	NO	NO	NO	YES	**YES**	YES
Day-of-Week FE	NO	NO	NO	YES	**YES**	YES
SE	Robust	Robust	Robust	Robust	**Day Clustered**	Newey
Marginal Effect	0.489	0.386	0.410	0.224	**0.224**	0.224
P-value	1.00e-08	1.00e-08	1.00e-08	1.00e-08	**1.00e-08**	1.00e-08

Robust standard errors in parentheses
*** p<0.01, ** p<0.05, * p<0.1

Supplementary Tab. 3: Coal responsiveness regression estimates for Czechia under alternative model specifications, with column (5) being our main preferred specification. The dependent variable is log-transformed coal generation The marginal effect, shown on the bottom, is our main estimate of interest, derived through the delta method from the Relative Price Coefficients, with its p-value listed below it. The statistical test used is a two-sided t-test. * indicates significance at the 10% level, ** indicates significance at the 5% level, and *** indicates significance at the 1% level.

Denmark						
	(1)	(2)	(3)	(4)	**(5)**	(6)
VARIABLES						
Relative Price	-0.22	0.87***	0.87***	1.28***	**1.28****	1.28***
	(0.135)	(0.131)	(0.131)	(0.137)	**(0.541)**	(0.190)
Relative Price Squared	0.26***	-0.18***	-0.18***	-0.38***	**-0.38***	-0.38***
	(0.054)	(0.053)	(0.053)	(0.054)	**(0.214)**	(0.075)
Relative Price Cubed	-0.03***	0.02**	0.02**	0.04***	**0.04**	0.04***
	(0.006)	(0.007)	(0.007)	(0.007)	**(0.026)**	(0.009)
Intermittent Renewables (GWh)		-0.15***	-0.15***	-0.16***	**-0.16*****	-0.16***
		(0.003)	(0.003)	(0.003)	**(0.010)**	(0.004)
Load (GWh)		0.17***	0.17***	0.09	**0.09**	0.09
		(0.064)	(0.064)	(0.070)	**(0.161)**	(0.095)
Load Squared (GWh)			0.01	0.01*	**0.01**	0.01
			(0.008)	(0.008)	**(0.016)**	(0.010)
Observations	10,223	10,223	10,223	10,223	**10,223**	10,223
Adjusted R-squared	0.193	0.373	0.373	0.553	**0.553**	
Month FE	NO	NO	YES	YES	**YES**	YES
Hour FE	NO	NO	NO	YES	**YES**	YES
Day-of-Week FE	NO	NO	NO	YES	**YES**	YES
SE	Robust	Robust	Robust	Robust	**Day Clustered**	Newey
Marginal Effect	0.375	0.383	0.383	0.302	**0.302**	0.302
P-value	1.00e-08	1.00e-08	1.00e-08	1.00e-08	**6.80e-06**	1.00e-08

Robust standard errors in parentheses
*** p<0.01, ** p<0.05, * p<0.1

Supplementary Tab. 4: Coal responsiveness regression estimates for Denmark under alternative model specifications, with column (5) being our main preferred specification. The dependent variable is log-transformed coal generation The marginal effect, shown on the bottom, is our main estimate of interest, derived through the delta method from the Relative Price Coefficients, with its p-value listed below it. The statistical test used is a two-sided t-test. * indicates significance at the 10% level, ** indicates significance at the 5% level, and *** indicates significance at the 1% level.

Germany						
	(1)	(2)	(3)	(4)	**(5)**	(6)
VARIABLES						
Relative Price	1.68***	2.40***	2.09***	2.43***	**2.43*** **	2.43***
	(0.102)	(0.068)	(0.072)	(0.075)	**(0.307)**	(0.105)
Relative Price Squared	-0.47***	-0.80***	-0.68***	-0.79***	**-0.79*** **	-0.79***
	(0.039)	(0.027)	(0.029)	(0.030)	**(0.122)**	(0.042)
Relative Price Cubed	0.04***	0.08***	0.07***	0.08***	**0.08*** **	0.08***
	(0.005)	(0.003)	(0.004)	(0.004)	**(0.015)**	(0.005)
Intermittent Renewables (GWh)		-0.02***	-0.02***	-0.02***	**-0.02*** **	-0.02***
		(0.000)	(0.000)	(0.000)	**(0.001)**	(0.000)
Nuclear (GWh)			-0.00***			
			(0.000)			
Load (GWh)		0.05***	0.05***	0.08***	**0.08*** **	0.08***
		(0.003)	(0.003)	(0.003)	**(0.008)**	(0.004)
Load Squared (GWh)			-0.00***	-0.00***	**-0.00*** **	-0.00***
			(0.000)	(0.000)	**(0.000)**	(0.000)
Observations	10,224	10,224	10,224	10,224	**10,224**	10,224
Adjusted R-squared	0.256	0.750	0.753	0.808	**0.808**	
Month FE	NO	NO	YES	YES	**YES**	YES
Hour FE	NO	NO	NO	YES	**YES**	YES
Day-of-Week FE	NO	NO	NO	YES	**YES**	YES
SE	Robust	Robust	Robust	Robust	**Day Clustered**	Newey
Marginal Effect	0.443	0.364	0.343	0.411	**0.411**	0.411
P-value	1.00e-08	1.00e-08	1.00e-08	1.00e-08	**1.00e-08**	1.00e-08

Robust standard errors in parentheses
*** p<0.01, ** p<0.05, * p<0.1

Supplementary Tab. 5: Coal responsiveness regression estimates for Germany under alternative model specifications, with column (5) being our main preferred specification. The dependent variable is log-transformed coal generation The marginal effect, shown on the bottom, is our main estimate of interest, derived through the delta method from the Relative Price Coefficients, with its p-value listed below it. The statistical test used is a two-sided t-test. * indicates significance at the 10% level, ** indicates significance at the 5% level, and *** indicates significance at the 1% level.

Finland						
	(1)	(2)	(3)	(4)	**(5)**	(6)
VARIABLES						
Relative Price	4.18***	4.56***	4.31***	3.27***	**3.27*** **	3.27***
	(0.216)	(0.171)	(0.175)	(0.108)	**(0.460)**	(0.149)
Relative Price Squared	-1.18***	-1.66***	-1.58***	-1.10***	**-1.10*** **	-1.10***
	(0.083)	(0.066)	(0.067)	(0.040)	**(0.168)**	(0.055)
Relative Price Cubed	0.11***	0.19***	0.18***	0.12***	**0.12*** **	0.12***
	(0.010)	(0.008)	(0.008)	(0.005)	**(0.019)**	(0.006)
Intermittent Renewables (GWh)		-0.08***	-0.06***	-0.06***	**-0.06*** **	-0.06***
		(0.008)	(0.008)	(0.005)	**(0.018)**	(0.007)
Nuclear (GWh)			0.00***			
			(0.000)			
Load (GWh)		1.48***	1.40***	0.70***	**0.70*** **	0.70***
		(0.056)	(0.057)	(0.041)	**(0.122)**	(0.056)
Load Squared (GWh)			-0.05***	-0.02***	**-0.02*** **	-0.02***
			(0.003)	(0.002)	**(0.005)**	(0.003)
Observations	8,882	8,882	8,882	8,882	**8,882**	8,882
Adjusted R-squared	0.319	0.543	0.545	0.824	**0.824**	
Month FE	NO	NO	YES	YES	**YES**	YES
Hour FE	NO	NO	NO	YES	**YES**	YES
Day-of-Week FE	NO	NO	NO	YES	**YES**	YES
SE	Robust	Robust	Robust	Robust	**Day Clustered**	Newey
Marginal Effect	1.015	0.467	0.433	0.463	**0.463**	0.463
P-value	1.00e-08	1.00e-08	1.00e-08	1.00e-08	**1.00e-08**	1.00e-08

Robust standard errors in parentheses
*** p<0.01, ** p<0.05, * p<0.1

Supplementary Tab. 6: Coal responsiveness regression estimates for Finland under alternative model specifications, with column (5) being our main preferred specification. The dependent variable is log-transformed coal generation The marginal effect, shown on the bottom, is our main estimate of interest, derived through the delta method from the Relative Price Coefficients, with its p-value listed below it. The statistical test used is a two-sided t-test. * indicates significance at the 10% level, ** indicates significance at the 5% level, and *** indicates significance at the 1% level.

	Greece					
	(1)	(2)	(3)	(4)	**(5)**	(6)
VARIABLES						
Relative Price	-0.74***	-1.38***	-1.38***	-0.24	**-0.24**	-0.24
	(0.160)	(0.155)	(0.155)	(0.188)	**(0.855)**	(0.263)
Relative Price Squared	0.29***	0.60***	0.60***	0.19**	**0.19**	0.19*
	(0.065)	(0.063)	(0.063)	(0.074)	**(0.336)**	(0.103)
Relative Price Cubed	-0.03***	-0.07***	-0.07***	-0.02***	**-0.02**	-0.02**
	(0.008)	(0.008)	(0.008)	(0.009)	**(0.040)**	(0.012)
Intermittent Renewables (GWh)		-0.11***	-0.11***	-0.06***	**-0.06*** **	-0.06***
		(0.005)	(0.005)	(0.006)	**(0.024)**	(0.008)
Load (GWh)		0.05**	0.05**	0.46***	**0.46*** **	0.46***
		(0.024)	(0.024)	(0.035)	**(0.087)**	(0.043)
Load Squared (GWh)			0.02***	-0.01***	**-0.01* **	-0.01***
			(0.002)	(0.003)	**(0.006)**	(0.003)
Observations	10,213	10,213	10,213	10,213	**10,213**	10,213
Adjusted R-squared	0.040	0.303	0.303	0.388	**0.388**	
Month FE	NO	NO	NO	NO	**NO**	NO
Hour FE	NO	NO	NO	YES	**YES**	YES
Day-of-Week FE	NO	NO	NO	YES	**YES**	YES
SE	Robust	Robust	Robust	Robust	**Day Clustered**	Newey
Marginal Effect	0.0813	0.0659	0.0659	0.179	**0.179**	0.179
P-value	1.00e-08	4.21e-08	4.21e-08	1.00e-08	**0.0159**	1.00e-08

Robust standard errors in parentheses
*** p<0.01, ** p<0.05, * p<0.1

Supplementary Tab. 7: Coal responsiveness regression estimates for Greece under alternative model specifications, with column (5) being our main preferred specification. The dependent variable is log-transformed coal generation The marginal effect, shown on the bottom, is our main estimate of interest, derived through the delta method from the Relative Price Coefficients, with its p-value listed below it. The statistical test used is a two-sided t-test. * indicates significance at the 10% level, ** indicates significance at the 5% level, and *** indicates significance at the 1% level.

Hungary						
	(1)	(2)	(3)	(4)	**(5)**	(6)
VARIABLES						
Relative Price	0.31***	0.34***	0.43***	0.09	**0.09**	0.09
	(0.075)	(0.074)	(0.076)	(0.069)	**(0.270)**	(0.094)
Relative Price Squared	-0.10***	-0.13***	-0.17***	-0.00	**-0.00**	-0.00
	(0.029)	(0.029)	(0.029)	(0.026)	**(0.102)**	(0.036)
Relative Price Cubed	0.01***	0.02***	0.02***	-0.00	**-0.00**	-0.00
	(0.003)	(0.003)	(0.004)	(0.003)	**(0.012)**	(0.004)
Intermittent Renewables (GWh)		-0.01	-0.01	0.06***	**0.06****	0.06***
		(0.006)	(0.006)	(0.011)	**(0.026)**	(0.014)
Nuclear (GWh)			-0.00***			
			(0.000)			
Load (GWh)		0.22***	0.23***	0.17***	**0.17****	0.17***
		(0.030)	(0.029)	(0.029)	**(0.077)**	(0.040)
Load Squared (GWh)			-0.02***	-0.01**	**-0.01**	-0.01
			(0.003)	(0.003)	**(0.007)**	(0.004)
Observations	10,210	10,210	10,210	10,210	**10,210**	10,210
Adjusted R-squared	0.008	0.041	0.048	0.139	**0.139**	
Month FE	NO	NO	YES	YES	**YES**	YES
Hour FE	NO	NO	NO	YES	**YES**	YES
Day-of-Week FE	NO	NO	NO	YES	**YES**	YES
SE	Robust	Robust	Robust	Robust	**Day Clustered**	Newey
Marginal Effect	0.0524	0.0263	0.0267	0.0454	**0.0454**	0.0454
P-value	1.00e-08	0.000381	0.000328	8.26e-08	**0.191**	0.000119

Robust standard errors in parentheses
*** p<0.01, ** p<0.05, * p<0.1

Supplementary Tab. 8: Coal responsiveness regression estimates for Hungary under alternative model specifications, with column (5) being our main preferred specification. The dependent variable is log-transformed coal generation The marginal effect, shown on the bottom, is our main estimate of interest, derived through the delta method from the Relative Price Coefficients, with its p-value listed below it. The statistical test used is a two-sided t-test. * indicates significance at the 10% level, ** indicates significance at the 5% level, and *** indicates significance at the 1% level.

Ireland						
	(1)	(2)	(3)	(4)	**(5)**	(6)
VARIABLES						
Relative Price	-2.02***	0.63***	0.63***	-2.11***	**-2.11*** **	-2.11***
	(0.171)	(0.132)	(0.132)	(0.219)	**(0.759)**	(0.283)
Relative Price Squared	1.03***	-0.03	-0.03	0.91***	**0.91*** **	0.91***
	(0.070)	(0.054)	(0.054)	(0.081)	**(0.276)**	(0.104)
Relative Price Cubed	-0.14***	-0.01	-0.01	-0.11***	**-0.11*** **	-0.11***
	(0.009)	(0.007)	(0.007)	(0.009)	**(0.032)**	(0.012)
Intermittent Renewables (GWh)		-0.32***	-0.32***	-0.33***	**-0.33*** **	-0.33***
		(0.004)	(0.004)	(0.005)	**(0.016)**	(0.006)
Load (GWh)		0.08***	0.08***	0.08***	**0.08***	0.08***
		(0.011)	(0.011)	(0.017)	**(0.050)**	(0.023)
Load Squared (GWh)			0.04***	0.01***	**0.01**	0.01**
			(0.002)	(0.004)	**(0.010)**	(0.005)
Observations	9,974	9,974	9,974	9,974	**9,974**	9,974
Adjusted R-squared	0.125	0.583	0.583	0.629	**0.629**	
Month FE	NO	NO	YES	YES	**YES**	YES
Hour FE	NO	NO	NO	YES	**YES**	YES
Day-of-Week FE	NO	NO	NO	YES	**YES**	YES
SE	Robust	Robust	Robust	Robust	**Day Clustered**	Newey
Marginal Effect	0.249	0.408	0.408	0.0295	**0.0295**	0.0295
P-value	1.00e-08	1.00e-08	1.00e-08	0.324	**0.797**	0.456

Robust standard errors in parentheses
*** p<0.01, ** p<0.05, * p<0.1

Supplementary Tab. 9: Coal responsiveness regression estimates for Ireland under alternative model specifications, with column (5) being our main preferred specification. The dependent variable is log-transformed coal generation The marginal effect, shown on the bottom, is our main estimate of interest, derived through the delta method from the Relative Price Coefficients, with its p-value listed below it. The statistical test used is a two-sided t-test. * indicates significance at the 10% level, ** indicates significance at the 5% level, and *** indicates significance at the 1% level.

Italy						
	(1)	(2)	(3)	(4)	**(5)**	(6)
VARIABLES						
Relative Price	4.30***	3.42***	3.42***	4.32***	**4.32*****	4.32***
	(0.125)	(0.123)	(0.123)	(0.135)	**(0.603)**	(0.190)
Relative Price Squared	-1.51***	-1.19***	-1.19***	-1.42***	**-1.42*****	-1.42***
	(0.053)	(0.051)	(0.051)	(0.053)	**(0.239)**	(0.075)
Relative Price Cubed	0.17***	0.14***	0.14***	0.15***	**0.15*****	0.15***
	(0.007)	(0.007)	(0.007)	(0.007)	**(0.030)**	(0.009)
Intermittent Renewables (GWh)		-0.03***	-0.03***	-0.04***	**-0.04*****	-0.04***
		(0.001)	(0.001)	(0.002)	**(0.005)**	(0.002)
Load (GWh)		0.02***	0.02***	0.04***	**0.04****	0.04***
		(0.004)	(0.004)	(0.005)	**(0.019)**	(0.008)
Load Squared (GWh)			0.00	-0.00***	**-0.00**	-0.00**
			(0.000)	(0.000)	**(0.000)**	(0.000)
Observations	10,224	10,224	10,224	10,224	**10,224**	10,224
Adjusted R-squared	0.314	0.407	0.407	0.529	**0.529**	
Month FE	NO	NO	YES	YES	**YES**	YES
Hour FE	NO	NO	NO	YES	**YES**	YES
Day-of-Week FE	NO	NO	NO	YES	**YES**	YES
SE	Robust	Robust	Robust	Robust	**Day Clustered**	Newey
Marginal Effect	0.630	0.499	0.499	0.747	**0.747**	0.747
P-value	1.00e-08	1.00e-08	1.00e-08	1.00e-08	**1.00e-08**	1.00e-08

Robust standard errors in parentheses
*** p<0.01, ** p<0.05, * p<0.1

Supplementary Tab. 10: Coal responsiveness regression estimates for Italy under alternative model specifications, with column (5) being our main preferred specification. The dependent variable is log-transformed coal generation The marginal effect, shown on the bottom, is our main estimate of interest, derived through the delta method from the Relative Price Coefficients, with its p-value listed below it. The statistical test used is a two-sided t-test. * indicates significance at the 10% level, ** indicates significance at the 5% level, and *** indicates significance at the 1% level.

Netherlands						
	(1)	(2)	(3)	(4)	**(5)**	(6)
VARIABLES						
Relative Price	0.96***	1.37***	0.77***	-1.88***	**-1.88*****	-1.88***
	(0.139)	(0.126)	(0.116)	(0.145)	**(0.642)**	(0.204)
Relative Price Squared	0.03	-0.18***	0.03	0.88***	**0.88*****	0.88***
	(0.056)	(0.050)	(0.047)	(0.056)	**(0.244)**	(0.078)
Relative Price Cubed	-0.03***	-0.00	-0.03***	-0.12***	**-0.12*****	-0.12***
	(0.007)	(0.006)	(0.006)	(0.007)	**(0.029)**	(0.009)
Intermittent Renewables (GWh)		-0.09***	-0.09***	-0.11***	**-0.11*****	-0.11***
		(0.004)	(0.004)	(0.003)	**(0.012)**	(0.005)
Nuclear (GWh)			0.00***			
			(0.000)			
Load (GWh)		0.26***	0.25***	0.27***	**0.27*****	0.27***
		(0.011)	(0.012)	(0.010)	**(0.023)**	(0.014)
Load Squared (GWh)			-0.01***	-0.01***	**-0.01*****	-0.01***
			(0.001)	(0.000)	**(0.001)**	(0.001)
Observations	10,206	10,206	10,206	10,206	**10,206**	10,206
Adjusted R-squared	0.318	0.463	0.496	0.731	**0.731**	
Month FE	NO	NO	YES	YES	**YES**	YES
Hour FE	NO	NO	NO	YES	**YES**	YES
Day-of-Week FE	NO	NO	NO	YES	**YES**	YES
SE	Robust	Robust	Robust	Robust	**Day Clustered**	Newey
Marginal Effect	0.728	0.654	0.558	0.0868	**0.0868**	0.0868
P-value	1.00e-08	1.00e-08	1.00e-08	1.30e-06	**0.280**	0.000565

Robust standard errors in parentheses
*** p<0.01, ** p<0.05, * p<0.1

Supplementary Tab. 11: Coal responsiveness regression estimates for Netherlands under alternative model specifications, with column (5) being our main preferred specification. The dependent variable is log-transformed coal generation The marginal effect, shown on the bottom, is our main estimate of interest, derived through the delta method from the Relative Price Coefficients, with its p-value listed below it. The statistical test used is a two-sided t-test. * indicates significance at the 10% level, ** indicates significance at the 5% level, and *** indicates significance at the 1% level.

Romania						
	(1)	(2)	(3)	(4)	**(5)**	(6)
VARIABLES						
Relative Price	-0.15***	-0.34***	-0.35***	0.13***	**0.13**	0.13**
	(0.049)	(0.042)	(0.044)	(0.049)	**(0.194)**	(0.067)
Relative Price Squared	0.07***	0.12***	0.12***	-0.04**	**-0.04**	-0.04*
	(0.019)	(0.017)	(0.017)	(0.019)	**(0.075)**	(0.026)
Relative Price Cubed	-0.01***	-0.01***	-0.01***	0.00**	**0.00**	0.00
	(0.002)	(0.002)	(0.002)	(0.002)	**(0.009)**	(0.003)
Intermittent Renewables (GWh)		-0.07***	-0.07***	-0.06***	**-0.06*** **	-0.06***
		(0.002)	(0.002)	(0.002)	**(0.006)**	(0.002)
Nuclear (GWh)			0.00			
			(0.000)			
Load (GWh)		0.35***	0.35***	0.43***	**0.43*** **	0.43***
		(0.016)	(0.016)	(0.016)	**(0.044)**	(0.022)
Load Squared (GWh)			-0.02***	-0.02***	**-0.02*** **	-0.02***
			(0.001)	(0.001)	**(0.003)**	(0.002)
Observations	10,173	10,173	10,173	10,173	**10,173**	10,173
Adjusted R-squared	0.004	0.285	0.285	0.490	**0.490**	
Month FE	NO	NO	YES	YES	**YES**	YES
Hour FE	NO	NO	NO	YES	**YES**	YES
Day-of-Week FE	NO	NO	NO	YES	**YES**	YES
SE	Robust	Robust	Robust	Robust	**Day Clustered**	Newey
Marginal Effect	0.00643	-0.0488	-0.0499	0.0206	**0.0206**	0.0206
P-value	0.151	1.00e-08	1.00e-08	0.000348	**0.381**	0.00976

Robust standard errors in parentheses
*** p<0.01, ** p<0.05, * p<0.1

Supplementary Tab. 12: Coal responsiveness regression estimates for Romania under alternative model specifications, with column (5) being our main preferred specification. The dependent variable is log-transformed coal generation The marginal effect, shown on the bottom, is our main estimate of interest, derived through the delta method from the Relative Price Coefficients, with its p-value listed below it. The statistical test used is a two-sided t-test. * indicates significance at the 10% level, ** indicates significance at the 5% level, and *** indicates significance at the 1% level.

Poland						
	(1)	(2)	(3)	(4)	(5)	(6)
VARIABLES						
Relative Price	0.19***	0.62***	0.62***	0.59***	0.59***	0.59***
	(0.061)	(0.024)	(0.024)	(0.023)	(0.078)	(0.023)
Relative Price Squared	-0.01	-0.21***	-0.21***	-0.19***	-0.19***	-0.19***
	(0.024)	(0.010)	(0.010)	(0.009)	(0.030)	(0.009)
Relative Price Cubed	-0.00	0.02***	0.02***	0.02***	0.02***	0.02***
	(0.003)	(0.001)	(0.001)	(0.001)	(0.003)	(0.001)
Intermittent Renewables (GWh)		-0.06***	-0.06***	-0.05***	-0.05***	-0.05***
		(0.000)	(0.000)	(0.000)	(0.001)	(0.000)
Load (GWh)		0.13***	0.13***	0.14***	0.14***	0.14***
		(0.003)	(0.003)	(0.003)	(0.009)	(0.003)
Load Squared (GWh)			-0.00***	-0.00***	-0.00***	-0.00***
			(0.000)	(0.000)	(0.000)	(0.000)
Observations	10,224	10,224	10,224	10,224	10,224	10,224
Adjusted R-squared	0.123	0.877	0.877	0.911	0.911	
Month FE	NO	NO	NO	YES	YES	YES
Hour FE	NO	NO	NO	YES	YES	YES
Day-of-Week FE	NO	NO	NO	YES	YES	YES
SE	Robust	Robust	Robust	Robust	Day Clustered	Newey
Marginal Effect	0.135	0.0908	0.0908	0.103	0.103	0.103

Robust standard errors in parentheses

*** p<0.01, ** p<0.05, * p<0.1

Supplementary Tab. 13: Coal responsiveness regression estimates for Poland under alternative model specifications, with column (5) being our main preferred specification. The dependent variable is log-transformed coal generation The marginal effect, shown on the bottom, is our main estimate of interest, derived through the delta method from the Relative Price Coefficients, with its p-value listed below it. The statistical test used is a two-sided t-test. * indicates significance at the 10% level, ** indicates significance at the 5% level, and *** indicates significance at the 1% level.

Country	Main (BIC)		Quadratic (BIC)		Linear (BIC)		Levels (BIC)	
Bulgaria	0.259	(-15781)	0.215	(-14788)	0.125	(-13896)	0.565	(371)
Czech Republic	0.224	(-10530)	0.199	(-10307)	0.141	(-10081)	0.627	(12335)
Germany	0.411	(-6633)	0.366	(-6169)	0.204	(-4991)	5.481	(44168)
Denmark	0.302	(7979)	0.281	(7994)	0.195	(8072)	0.198	(-2926)
Spain	0.705	(4722)	0.612	(5298)	0.322	(6485)	0.503	(-2141)
Finland	0.463	(7641)	0.419	(7850)	0.256	(8181)	0.151	(-19702)
Greece	0.209	(12839)	0.209	(12839)	0.200	(12831)	0.072	(-701)
Croatia	0.084	(-8001)	0.069	(-8004)	0.006	(-7936)	0.110	(-30657)
Hungary	0.045	(-2380)	0.047	(-2389)	0.013	(-2360)	0.006	(-26844)
Italy	0.747	(5517)	0.656	(6002)	0.413	(6818)	1.205	(14661)
Ireland	0.029	(7713)	0.162	(7863)	0.178	(7855)	0.040	(-12084)
Netherlands	0.087	(7061)	0.153	(7310)	0.107	(7327)	0.338	(16598)
Poland	0.103	(-27843)	0.092	(-27644)	0.049	(-26997)	1.322	(22270)
Romania	0.021	(-15124)	0.018	(-15130)	0.008	(-15129)	0.002	(-11768)
**Average**	**0.264**		**0.206**		**0.155**		**0.436**	

Supplementary Tab. 14: Coal responsiveness regression estimates under different functional forms. The marginal estimate (derived through the delta method) is shown for each country, with the BIC in parenthesis. A more negative BIC indicates better fit. The main functional form includes a cubic, quadratic, and linear term of the relative price. The quadratic column includes a quadratic and a linear term, and the linear column includes only a linear term. The levels column is the same as the main regression but, in contrast to the others, the dependent variable of coal generation is in levels rather than logs. The sample size for each country is shown in Extended Data Fig. 5.

Country	January 2021-<br>January 2022	April 2021-<br>November 2021	April 2021-<br>January 2022	April 2021-<br>March 2022	(Main Spec.)<br>April 2021-<br>June 2022
Bulgaria	0.307***	0.602***	0.277***	0.203***	0.259***
Czech Republic	-0.0414	-0.0421	-0.0224	0.0197	0.224***
Germany	0.295***	0.608***	0.292***	0.259***	0.411***
Denmark	-0.0137	0.145	0.0337	0.00214	0.302***
Spain	-0.174	-0.279	-0.184	-0.163	0.705***
Finland	0.289	-0.426	0.251	0.265**	0.463***
Greece	1.449***	2.034***	0.451***	-0.172*	0.179***
Croatia	0.0231	0.0923	0.0141	0.0258	0.0836*
Hungary	0.420***	1.086***	0.380*	0.323***	0.0454
Italy	1.369***	1.742***	1.164***	0.825***	0.747***
Ireland	-0.113	0.0374	-0.0685	0.150	0.0295
Netherlands	0.109	0.0657	0.104	0.0882	0.0868
Poland	0.0904***	0.156***	0.0894***	0.0834***	0.103***
Romania	-0.192	-0.0937	-0.191	-0.0723*	0.0206
**Average**	0.301	0.351	0.161	0.142	0.265

Supplementary Tab. 15: Main (marginal) coal responsiveness estimate shown for each country under different time periods, using the main regression specification. The statistical test used is a two-sided t-test. * indicates significance at the 10% level, ** indicates significance at the 5% level, and *** indicates significance at the 1% level. More detailed regression results for each time period are shown in Supp. Tab. 16-19.

	April 2021 - January 2022													
	(1)	(2)	(3)	(4)	(5)	(6)	(7)	(8)	(9)	(10)	(11)	(12)	(13)	(14)
VARIABLES	BG	CZ	DE	DK	ES	FI	GR	HR	HU	IT	IE	NL	PL	RO
Relative Price	1.520***	-0.696*	1.262**	-0.891	-1.557**	1.406	1.354	-0.008	2.195***	5.689***	-1.108	0.064	0.499***	-0.574*
	(0.383)	(0.411)	(0.529)	(1.063)	(0.764)	(1.139)	(1.005)	(0.384)	(0.760)	(1.406)	(0.882)	(0.939)	(0.153)	(0.311)
Relative Price Squared	-0.495***	0.273*	-0.363*	0.394	0.588**	-0.449	-0.348	0.024	-0.708***	-1.791***	0.427	0.060	-0.160***	0.133
	(0.136)	(0.148)	(0.193)	(0.360)	(0.252)	(0.375)	(0.355)	(0.142)	(0.259)	(0.487)	(0.309)	(0.322)	(0.051)	(0.112)
Relative Price Cubed	0.052***	-0.030*	0.033	-0.047	-0.071***	0.047	0.034	-0.006	0.071**	0.186***	-0.048	-0.016	0.016***	-0.010
	(0.015)	(0.016)	(0.022)	(0.039)	(0.027)	(0.040)	(0.040)	(0.016)	(0.028)	(0.054)	(0.034)	(0.035)	(0.005)	(0.013)
Intermittent Renewables (GWh)	-0.135***	-0.067***	-0.024***	-0.143***	-0.024***	-0.112***	-0.073***	-0.056**	0.091**	-0.025***	-0.325***	-0.103***	-0.053***	-0.043***
	(0.023)	(0.021)	(0.001)	(0.013)	(0.003)	(0.021)	(0.028)	(0.027)	(0.035)	(0.006)	(0.017)	(0.013)	(0.002)	(0.006)
Load (GWh)	0.279***	0.246***	0.104***	0.268	0.109***	1.152***	0.189**	0.362**	0.141	-0.019	-0.278***	0.269***	0.157***	0.339***
	(0.057)	(0.049)	(0.009)	(0.183)	(0.031)	(0.135)	(0.090)	(0.143)	(0.097)	(0.014)	(0.039)	(0.040)	(0.010)	(0.054)
Load Squared (GWh)	-0.016***	-0.008***	-0.001***	0.001	-0.002***	-0.040***	0.007	-0.056**	-0.003	0.001***	0.071***	-0.010***	-0.002***	-0.018***
	(0.005)	(0.003)	(0.000)	(0.018)	(0.000)	(0.006)	(0.006)	(0.028)	(0.009)	(0.000)	(0.010)	(0.002)	(0.000)	(0.003)
Observations	7,344	7,344	7,344	7,344	7,326	6,002	7,344	5,847	7,330	7,344	7,240	7,326	7,344	7,293
R-squared	0.912	0.818	0.822	0.565	0.683	0.848	0.320	0.322	0.155	0.535	0.579	0.751	0.914	0.542
Month FE	YES	YES	YES	YES	YES	YES	YES	YES	YES	YES	YES	YES	YES	YES
Hour FE	YES	YES	YES	YES	YES	YES	YES	YES	YES	YES	YES	YES	YES	YES
Day of Week FE	YES	YES	YES	YES	YES	YES	YES	YES	YES	YES	YES	YES	YES	YES
**Marginal Effect**	**0.277**	**-0.0224**	**0.292**	**0.0337**	**-0.184**	**0.251**	**0.451**	**0.0141**	**0.380**	**1.164**	**-0.0685**	**0.104**	**0.0894**	**-0.191**
Robust standard errors in parentheses														
*** p<0.01, ** p<0.05, * p<0.1														

Supplementary Tab. 16: Estimates of main regression results of responsiveness of log-transformed coal generation factor to relative price (natural gas price/coal price), during April 1 2021-December 31 2021. The marginal effect, which is the estimate of interest, is derived through the delta method from the relative price coefficients, using the mean value of relative price during this period. Standard errors are clustered at the daily level, which is the unit of variation of the treatment variable. The statistical test used is the two-sided t-test. * indicates significance at the 10% level, ** indicates significance at the 5% level, and *** indicates significance at the 1% level.

	January 2021 - December 2021													
	(1)	(2)	(3)	(4)	(5)	(6)	(7)	(8)	(9)	(10)	(11)	(12)	(13)	(14)
VARIABLES	BG	CZ	DE	DK	ES	FI	GR	HR	HU	IT	IE	NL	PL	RO
Relative Price	1.389***	-0.773*	1.259**	-0.837	-1.225	1.438	4.498***	-0.029	2.209***	5.795***	-1.205	-0.008	0.477***	-0.617**
	(0.385)	(0.406)	(0.525)	(1.074)	(0.754)	(1.162)	(1.057)	(0.381)	(0.764)	(1.372)	(0.887)	(0.955)	(0.153)	(0.310)
Relative Price Squared	-0.434***	0.302**	-0.369*	0.346	0.464*	-0.451	-1.155***	0.036	-0.708***	-1.758***	0.452	0.094	-0.153***	0.156
	(0.135)	(0.148)	(0.195)	(0.364)	(0.244)	(0.379)	(0.360)	(0.141)	(0.259)	(0.468)	(0.311)	(0.327)	(0.051)	(0.111)
Relative Price Cubed	0.045***	-0.033*	0.034	-0.039	-0.057**	0.047	0.105**	-0.007	0.071**	0.178***	-0.050	-0.020	0.015***	-0.013
	(0.015)	(0.017)	(0.022)	(0.039)	(0.026)	(0.040)	(0.040)	(0.016)	(0.028)	(0.052)	(0.034)	(0.035)	(0.005)	(0.012)
Intermittent Renewables (GWh)	-0.138***	-0.071***	-0.025***	-0.126***	-0.027***	-0.112***	-0.063**	-0.037	0.087**	-0.022***	-0.332***	-0.101***	-0.055***	-0.043***
	(0.026)	(0.022)	(0.001)	(0.013)	(0.003)	(0.027)	(0.028)	(0.025)	(0.040)	(0.006)	(0.019)	(0.015)	(0.002)	(0.007)
Load (GWh)	0.299***	0.250***	0.101***	0.379*	0.094***	1.120***	0.244**	0.343**	0.044	-0.039***	-0.281***	0.270***	0.164***	0.302***
	(0.070)	(0.051)	(0.009)	(0.193)	(0.035)	(0.138)	(0.095)	(0.159)	(0.122)	(0.015)	(0.044)	(0.042)	(0.011)	(0.063)
Load Squared (GWh)	-0.022***	-0.010***	-0.001***	-0.014	-0.001**	-0.038***	0.004	-0.067**	0.006	0.001***	0.073***	-0.010***	-0.003***	-0.015***
	(0.006)	(0.003)	(0.000)	(0.019)	(0.001)	(0.006)	(0.006)	(0.031)	(0.012)	(0.000)	(0.011)	(0.002)	(0.000)	(0.004)
Observations	6,600	6,600	6,600	6,600	6,583	5,258	6,600	5,103	6,586	6,600	6,496	6,582	6,600	6,549
R-squared	0.907	0.810	0.826	0.557	0.660	0.836	0.409	0.348	0.145	0.544	0.559	0.748	0.912	0.537
Month FE	YES	YES	YES	YES	YES	YES	YES	YES	YES	YES	YES	YES	YES	YES
Hour FE	YES	YES	YES	YES	YES	YES	YES	YES	YES	YES	YES	YES	YES	YES
Day of Week FE	YES	YES	YES	YES	YES	YES	YES	YES	YES	YES	YES	YES	YES	YES
**Marginal Effect**	**0.307**	**-0.0414**	**0.295**	**-0.0137**	**-0.174**	**0.289**	**1.449**	**0.0231**	**0.420**	**1.369**	**-0.113**	**0.109**	**0.0904**	**-0.192**
Robust standard errors in parentheses														
*** p<0.01, ** p<0.05, * p<0.1														

Supplementary Tab. 17: Estimates of main regression results of responsiveness of log-transformed coal generation factor to relative price (natural gas price/coal price), during January 1 2021-December 31 2021. The marginal effect, which is the estimate of interest, is derived through the delta method from the relative price coefficients, using the mean value of relative price during this period. Standard errors are clustered at the daily level, which is the unit of variation of the treatment variable. The statistical test used is the two-sided t-test. * indicates significance at the 10% level, ** indicates significance at the 5% level, and *** indicates significance at the 1% level.

	April 2021 - March 2022													
	(1)	(2)	(3)	(4)	(5)	(6)	(7)	(8)	(9)	(10)	(11)	(12)	(13)	(14)
VARIABLES	BG	CZ	DE	DK	ES	FI	GR	HR	HU	IT	IE	NL	PL	RO
Relative Price	1.352***	-0.123	1.336***	-0.926	-2.074***	1.459	-1.825*	-0.017	1.956***	4.889***	-0.661	-1.016	0.461***	-0.530*
	(0.334)	(0.398)	(0.488)	(0.951)	(0.791)	(0.939)	(0.932)	(0.327)	(0.652)	(1.304)	(0.903)	(0.907)	(0.139)	(0.295)
Relative Price Squared	-0.459***	0.050	-0.407**	0.386	0.806***	-0.454	0.643*	0.034	-0.628***	-1.620***	0.360	0.508	-0.144***	0.183*
	(0.119)	(0.142)	(0.173)	(0.324)	(0.270)	(0.317)	(0.350)	(0.117)	(0.224)	(0.457)	(0.306)	(0.321)	(0.047)	(0.104)
Relative Price Cubed	0.050***	-0.004	0.039**	-0.045	-0.097***	0.047	-0.066	-0.007	0.063***	0.175***	-0.047	-0.069*	0.014***	-0.020*
	(0.013)	(0.016)	(0.019)	(0.035)	(0.029)	(0.034)	(0.041)	(0.013)	(0.024)	(0.051)	(0.033)	(0.036)	(0.005)	(0.012)
Intermittent Renewables (GWh)	-0.129***	-0.062***	-0.024***	-0.159***	-0.020***	-0.120***	-0.074***	-0.059**	0.082***	-0.024***	-0.329***	-0.096***	-0.053***	-0.043***
	(0.018)	(0.017)	(0.001)	(0.011)	(0.003)	(0.016)	(0.025)	(0.023)	(0.030)	(0.005)	(0.016)	(0.011)	(0.001)	(0.005)
Load (GWh)	0.321***	0.208***	0.092***	0.161	0.098***	1.102***	0.152*	0.329**	0.169*	-0.014	-0.320***	0.265***	0.154***	0.361***
	(0.049)	(0.045)	(0.008)	(0.167)	(0.029)	(0.131)	(0.086)	(0.133)	(0.086)	(0.014)	(0.035)	(0.030)	(0.009)	(0.053)
Load Squared (GWh)	-0.020***	-0.005**	-0.001***	0.011	-0.001***	-0.038***	0.008	-0.051**	-0.006	0.000**	0.082***	-0.010***	-0.002***	-0.019***
	(0.004)	(0.002)	(0.000)	(0.017)	(0.000)	(0.006)	(0.006)	(0.026)	(0.008)	(0.000)	(0.009)	(0.001)	(0.000)	(0.003)
Observations	8,759	8,760	8,760	8,759	8,729	7,418	8,758	6,988	8,746	8,760	8,628	8,742	8,760	8,709
R-squared	0.919	0.821	0.818	0.600	0.657	0.856	0.312	0.277	0.156	0.598	0.559	0.743	0.916	0.520
Month FE	YES	YES	YES	YES	YES	YES	YES	YES	YES	YES	YES	YES	YES	YES
Hour FE	YES	YES	YES	YES	YES	YES	YES	YES	YES	YES	YES	YES	YES	YES
Day of Week FE	YES	YES	YES	YES	YES	YES	YES	YES	YES	YES	YES	YES	YES	YES
**Marginal Effect**	**0.203**	**0.0197**	**0.259**	**0.00214**	**-0.163**	**0.265**	**-0.172**	**0.0258**	**0.323**	**0.825**	**0.150**	**0.0882**	**0.0834**	**-0.0723**

Robust standard errors in parentheses

*** p<0.01, ** p<0.05, * p<0.1

Supplementary Tab. 18: Estimates of main regression results of responsiveness of log-transformed coal generation factor to relative price (natural gas price/coal price), during April 1 2021-March 1 2022. The marginal effect, which is the estimate of interest, is derived through the delta method from the relative price coefficients, using the mean value of relative price during this period. Standard errors are clustered at the daily level, which is the unit of variation of the treatment variable. The statistical test used is the two-sided t-test. * indicates significance at the 10% level, ** indicates significance at the 5% level, and *** indicates significance at the 1% level.

April 2021 - November 2021														
	(1)	(2)	(3)	(4)	(5)	(6)	(7)	(8)	(9)	(10)	(11)	(12)	(13)	(14)
VARIABLES	BG	CZ	DE	DK	ES	FI	GR	HR	HU	IT	IE	NL	PL	RO
Relative Price	5.332***	-0.222	6.427***	2.839	-2.965	-8.630	6.334	1.534	11.992***	6.457*	1.708	-0.092	1.596***	1.108
	(1.503)	(1.561)	(1.625)	(4.132)	(2.345)	(5.779)	(4.229)	(1.361)	(3.230)	(3.588)	(3.387)	(3.893)	(0.530)	(1.251)
Relative Price Squared	-2.292***	0.093	-2.896***	-1.431	1.323	4.282	-1.490	-0.632	-5.487***	-1.599	-0.944	-0.001	-0.723***	-0.606
	(0.682)	(0.729)	(0.753)	(1.973)	(1.175)	(2.601)	(2.050)	(0.638)	(1.488)	(1.751)	(1.607)	(1.797)	(0.254)	(0.582)
Relative Price Cubed	0.325***	-0.014	0.427***	0.231	-0.192	-0.676*	0.077	0.081	0.823***	0.071	0.164	0.018	0.108***	0.091
	(0.101)	(0.111)	(0.115)	(0.304)	(0.187)	(0.384)	(0.325)	(0.097)	(0.224)	(0.275)	(0.251)	(0.271)	(0.039)	(0.089)
Intermittent Renewables (GWh)	-0.130***	-0.054**	-0.027***	-0.118***	-0.025***	-0.128***	-0.068**	-0.048**	0.020	-0.022***	-0.348***	-0.109***	-0.057***	-0.045***
	(0.031)	(0.027)	(0.001)	(0.016)	(0.004)	(0.030)	(0.030)	(0.024)	(0.050)	(0.007)	(0.022)	(0.017)	(0.002)	(0.008)
Load (GWh)	0.425***	0.134***	0.097***	0.425*	0.121***	1.110***	0.270**	0.333*	-0.103	-0.052***	-0.283***	0.261***	0.169***	0.299***
	(0.086)	(0.051)	(0.009)	(0.247)	(0.038)	(0.221)	(0.105)	(0.192)	(0.148)	(0.017)	(0.057)	(0.044)	(0.013)	(0.079)
Load Squared (GWh)	-0.040***	-0.001	-0.001***	-0.021	-0.001**	-0.038***	0.003	-0.070*	0.014	0.001***	0.076***	-0.010***	-0.003***	-0.015***
	(0.009)	(0.003)	(0.000)	(0.026)	(0.001)	(0.011)	(0.007)	(0.036)	(0.015)	(0.000)	(0.015)	(0.002)	(0.000)	(0.005)
Observations	5,856	5,856	5,856	5,856	5,842	4,514	5,856	4,359	5,842	5,856	5,765	5,838	5,856	5,805
R-squared	0.904	0.782	0.834	0.423	0.596	0.798	0.435	0.143	0.168	0.526	0.563	0.725	0.909	0.551
Month FE	YES	YES	YES	YES	YES	YES	YES	YES	YES	YES	YES	YES	YES	YES
Hour FE	YES	YES	YES	YES	YES	YES	YES	YES	YES	YES	YES	YES	YES	YES
Day of Week FE	YES	YES	YES	YES	YES	YES	YES	YES	YES	YES	YES	YES	YES	YES
**Marginal Effect**	**0.602**	**-0.0421**	**0.608**	**0.145**	**-0.279**	**-0.426**	**2.034**	**0.0923**	**1.086**	**1.742**	**0.0374**	**0.0657**	**0.156**	**-0.0937**
Robust standard errors in parentheses														
*** p<0.01, ** p<0.05, * p<0.1														

Supplementary Tab. 19: Estimates of main regression results of responsiveness of log-transformed coal generation factor to relative price (natural gas price/coal price), during April 1 2021-October 31 2021. The marginal effect, which is the estimate of interest, is derived through the delta method from the relative price coefficients, using the mean value of relative price during this period. Standard errors are clustered at the daily level, which is the unit of variation of the treatment variable. The statistical test used is the two-sided t-test. * indicates significance at the 10% level, ** indicates significance at the 5% level, and *** indicates significance at the 1% level.

VARIABLES	(1) BG	(2) CZ	(3) DE	(4) DK	(5) ES	(6) FI	(7) GR	(8) HR	(9) HU	(10) IT	(11) IE	(12) NL	(13) PL	(14) RO
Relative Price	1.942***	1.131***	2.189***	1.103**	4.190***	3.199***	-0.440	0.493	0.166	4.159***	-2.548***	-2.124***	0.532***	0.060
	(0.251)	(0.271)	(0.290)	(0.531)	(0.567)	(0.463)	(0.860)	(0.423)	(0.277)	(0.599)	(0.776)	(0.698)	(0.078)	(0.196)
Relative Price Day Lagged	-0.001	0.098***	0.164***	0.119	0.258**	0.057	0.329**	0.095**	-0.048	0.132*	0.209***	0.176**	0.032***	0.025
	(0.023)	(0.035)	(0.048)	(0.091)	(0.117)	(0.064)	(0.130)	(0.038)	(0.044)	(0.071)	(0.079)	(0.084)	(0.011)	(0.027)
Intermittent Renewables (GWh)	-0.151***	-0.056***	-0.024***	-0.157***	-0.024***	-0.063***	-0.063***	-0.066***	0.057**	-0.040***	-0.336***	-0.111***	-0.055***	-0.056***
	(0.019)	(0.017)	(0.001)	(0.010)	(0.004)	(0.018)	(0.024)	(0.023)	(0.026)	(0.005)	(0.016)	(0.012)	(0.001)	(0.006)
Load (GWh)	0.313***	0.123***	0.076***	0.081	0.041	0.690***	0.447***	0.229**	0.171**	0.042**	0.081	0.263***	0.135***	0.431***
	(0.045)	(0.044)	(0.008)	(0.162)	(0.034)	(0.123)	(0.086)	(0.112)	(0.076)	(0.018)	(0.050)	(0.023)	(0.009)	(0.045)
Load Squared (GWh)	-0.020***	-0.001	-0.000***	0.015	-0.000	-0.021***	-0.011*	-0.033	-0.006	-0.000	0.013	-0.010***	-0.002***	-0.023***
	(0.004)	(0.002)	(0.000)	(0.016)	(0.001)	(0.006)	(0.006)	(0.022)	(0.007)	(0.000)	(0.010)	(0.001)	(0.000)	(0.003)
Observations	10,199	10,200	10,200	10,199	10,169	8,858	10,189	7,957	10,186	10,224	9,950	10,182	10,200	10,149
R-squared	0.896	0.784	0.812	0.556	0.551	0.825	0.396	0.218	0.143	0.533	0.635	0.734	0.912	0.497
Month FE	YES	YES	YES	YES	YES	YES	YES	YES	YES	YES	YES	YES	YES	YES
Hour FE	YES	YES	YES	YES	YES	YES	YES	YES	YES	YES	YES	YES	YES	YES
Day of Week FE	YES	YES	YES	YES	YES	YES	YES	YES	YES	YES	YES	YES	YES	YES
**Marginal Effect**	**0.255*****	**0.227*****	**0.417*****	**0.308*****	**0.713*****	**0.467*****	**0.196****	**0.113***	**0.0432**	**0.758*****	**0.0218**	**0.0994**	**0.103*****	**0.0166**
SE	Day clustered	Day Clustered	Day Clustered	Day Clustered	Day Clustered	Day Clustered	Day clustered	Day Clustered	Day Clustered	Day Clustered	Day Clustered	Day Clustered	Day Clustered	Day Clustered

Supplementary Tab. 20: Estimates of main regression results of responsiveness of log-transformed coal generation factor to relative price (natural gas price/coal price), including a lagged relative price regressor (1 day; 24 hours). The marginal effect, which is the estimate of interest, is derived through the delta method from the relative price coefficients, using the mean value of relative price during this period. Standard errors are clustered at the daily level, which is the unit of variation of the treatment variable. The statistical test used is the two-sided t-test. * indicates significance at the 10% level, ** indicates significance at the 5% level, and *** indicates significance at the 1% level.

Pooled Regression	
	Log Coal Generation
Relative Price	0.696
	(0.768)
Intermittent Renewables (GWh)	-0.024***
	(0.003)
Load (GWh)	0.082**
	(0.031)
Load Squared (GWh)	-0.001*
	(0.000)
**Marginal Effect**	**0.212****
	(0.0917)
Observations	139,174
Number of Countries	14
R-squared	0.313
Country FE	YES
Month FE	YES
Hour FE	YES
Day of Week FE	YES
SE	Day Clustered

Supplementary Tab. 21: Estimates of pooled regression results of responsiveness of log-transformed coal generation factor to relative price (natural gas price/coal price), for the entire dataset of countries, including country fixed effects. The marginal effect, which is the estimate of interest, is derived through the delta method from the relative price coefficients, using the mean value of relative price during this period. Standard errors are clustered at the daily level, which is the unit of variation of the treatment variable. The statistical test used is the two-sided t-test. * indicates significance at the 10% level, ** indicates significance at the 5% level, and *** indicates significance at the 1% level.

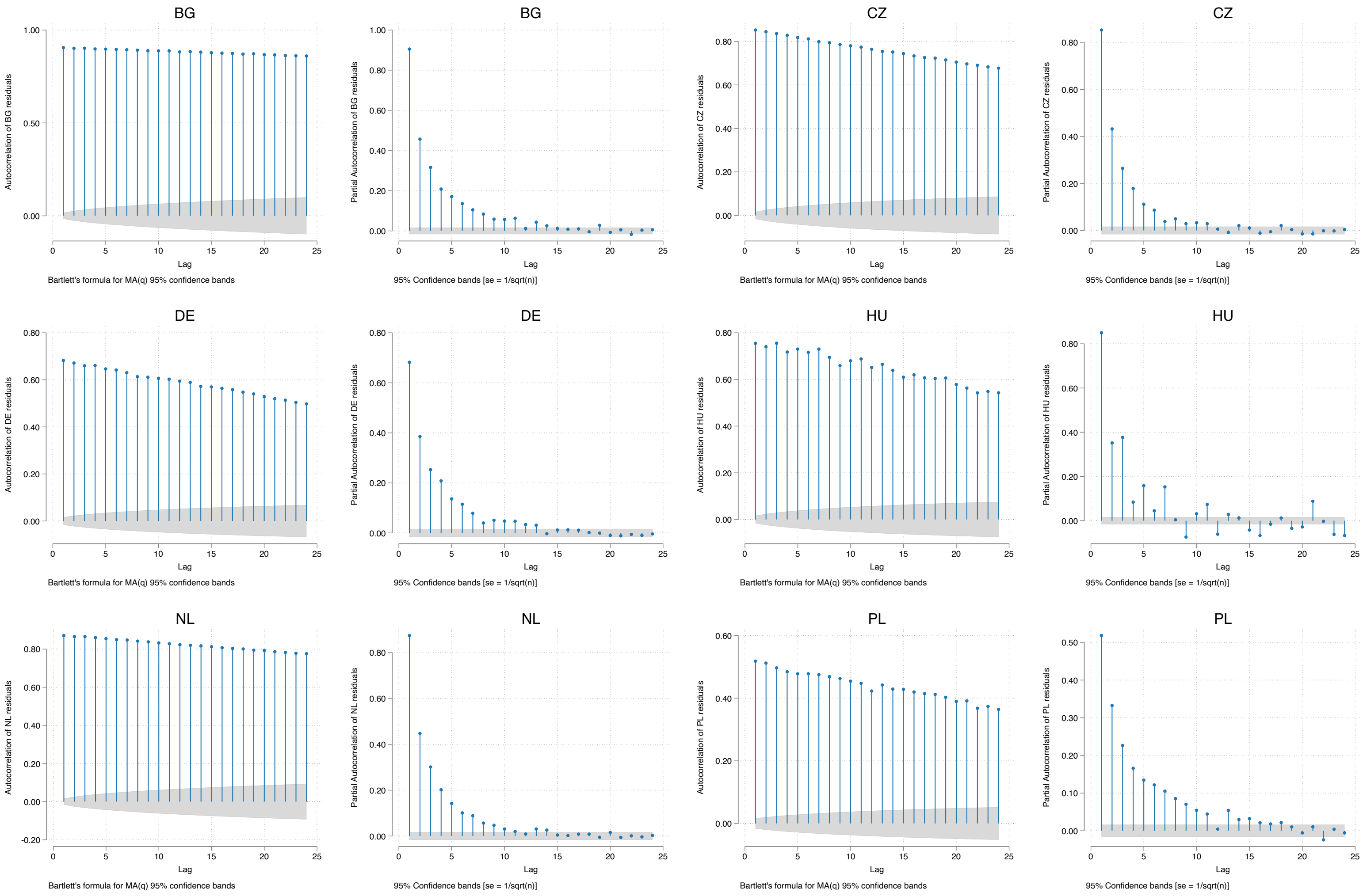

Supplementary Tab. 22: Autocorrelation (ACF; left) and Partial Autocorrelation (PACF; right) of the main regression residuals for six countries. These display clear intraday serial dependence, justifying the choice of clustered standard errors at the day level. The rapid decay of the PACF after the first few hours reflects the appropriate generation dynamics of hourly fuel-switching.

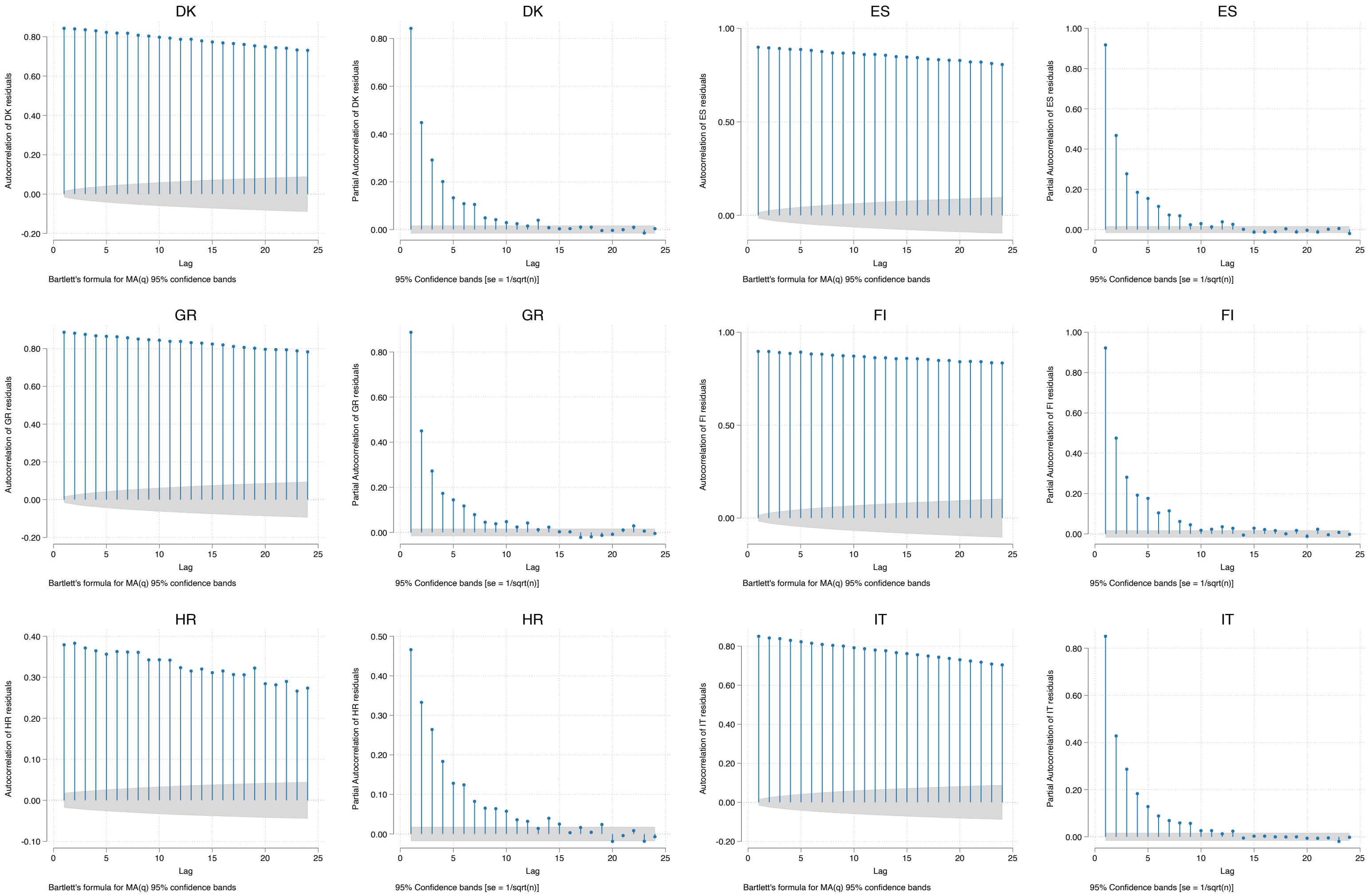

Supplementary Tab. 23: Autocorrelation (ACF; left) and Partial Autocorrelaiton (PACF; right) of the main regression residuals for six countries. These display clear intraday serial dependence, justifying the choice of clustered standard errors at the day level. The rapid decay of the PACF after the first few hours reflects the appropriate generation dynamics of hourly fuel-switching.

Pooled Regression (with ETS Price)	
	Log Coal Generation
Relative Price	0.882
	(0.513)
ETS Carbon Price	-0.001
	(0.002)
Intermittent Renewables (GWh)	-0.024***
	(0.003)
Load (GWh)	0.082**
	(0.031)
Load Squared (GWh)	-0.001*
	(0.000)
**Marginal Effect**	**0.234*****
	(0.0722)
Observations	139,174
Number of Countries	14
R-squared	0.313
Country FE	YES
Month FE	YES
Hour FE	YES
Day of Week FE	YES
SE	Day Clustered

Supplementary Tab. 24: Estimates of pooled regression results of responsiveness of log-transformed coal generation factor to relative price (natural gas price/coal price), for the entire dataset of countries, including carbon ETS price and country fixed effects. The marginal effect, which is the estimate of interest, is derived through the delta method from the relative price coefficients, using the mean value of relative price during this period. Standard errors are clustered at the daily level, which is the unit of variation of the treatment variable. The statistical test used is the two-sided t-test. * indicates significance at the 10% level, ** indicates significance at the 5% level, and *** indicates significance at the 1% level.

	(1) BG	(2) CZ	(3) DE	(4) DK	(5) ES	(6) FI	(7) GR	(8) HR	(9) HU	(10) IT	(11) IE	(12) NL	(13) PL	(14) RO
Relative Price	1.111***	0.554***	1.083***	0.281	1.833***	0.783***	-0.307	3.202***	-0.032	1.710***	-0.638*	-0.755***	0.349***	-0.010
	(0.141)	(0.145)	(0.112)	(0.187)	(0.278)	(0.140)	(0.232)	(0.602)	(0.141)	(0.258)	(0.368)	(0.226)	(0.043)	(0.130)
Relative Price Squared	-0.387***	-0.186***	-0.351***	-0.065	-0.593***	-0.267***	0.138	-1.048***	0.028	-0.562***	0.298**	0.384***	-0.111***	0.006
	(0.055)	(0.060)	(0.045)	(0.077)	(0.110)	(0.055)	(0.096)	(0.233)	(0.054)	(0.105)	(0.132)	(0.094)	(0.016)	(0.050)
Relative Price Cubed	0.043***	0.022***	0.037***	0.007	0.060***	0.030***	-0.016	0.109***	-0.005	0.061***	-0.037**	-0.052***	0.011***	-0.001
	(0.007)	(0.007)	(0.006)	(0.010)	(0.014)	(0.006)	(0.012)	(0.028)	(0.006)	(0.013)	(0.015)	(0.012)	(0.002)	(0.006)
IRE (GWh)	-0.102***	-0.030***	-0.012***	-0.061***	-0.010***	-0.029***	-0.028***	-0.086	0.030**	-0.017***	-0.124***	-0.043***	-0.033***	-0.035***
	(0.011)	(0.008)	(0.000)	(0.004)	(0.002)	(0.005)	(0.007)	(0.054)	(0.015)	(0.002)	(0.007)	(0.005)	(0.001)	(0.004)
Load (GWh)	0.118***	0.011	0.032***	-0.040	0.025***	-0.105***	0.090***	0.261	0.102**	0.019***	0.024	0.057***	0.048***	0.167***
	(0.026)	(0.023)	(0.003)	(0.050)	(0.009)	(0.024)	(0.026)	(0.197)	(0.041)	(0.007)	(0.027)	(0.009)	(0.004)	(0.006)
Load Squared (GWh)	-0.003	0.003**	-0.000***	0.014**	-0.000**	0.010***	0.002	-0.040	-0.004	-0.000	0.011**	-0.001***	-0.000***	-0.007***
	(0.002)	(0.001)	(0.000)	(0.005)	(0.000)	(0.001)	(0.002)	(0.038)	(0.004)	(0.000)	(0.005)	(0.000)	(0.000)	(0.001)
Marginal Effect	0.154	0.106	0.191	0.113	0.303	0.127	0.028	0.531	0.011	0.310	0.053	0.087	0.062	0.001
	(0.018)	(0.014)	(0.012)	(0.020)	(0.041)	(0.017)	(0.015)	(0.078)	(0.018)	(0.028)	(0.054)	(0.024)	(0.006)	(0.016)
Observations	10,224	10,224	10,224	10,224	10,224	10,224	10,224	10,224	10,224	10,224	10,224	10,224	10,224	10,224
R-squared	0.888	0.815	0.870	0.628	0.523	0.887	0.429	0.567	0.187	0.552	0.554	0.752	0.929	0.582
Month FE	YES	YES	YES	YES	YES	YES	YES	YES	YES	YES	YES	YES	YES	YES
Hour FE	YES	YES	YES	YES	YES	YES	YES	YES	YES	YES	YES	YES	YES	YES
Day of Week FE	YES	YES	YES	YES	YES	YES	YES	YES	YES	YES	YES	YES	YES	YES

Clustered standard errors on day in parentheses

*** p<0.01, ** p<0.05, * p<0.1

Supplementary Tab. 25: Estimates of regression results for an alternative dependent variable of coal capacity factor (generation/installed capacity) responsiveness to relative price (natural gas price/coal price). Marginal effect derived through the delta method from the relative price coefficients is our estimate of interest. The statistical test used is the two-sided t-test. * indicates significance at the 10% level, ** indicates significance at the 5% level, and *** indicates significance at the 1% level.

1.3.2 Econometric Model of Pass-Through

To examine the robustness our pass-through estimate results that we use for the policy counterfactual analysis over the period of 2022, we shorten the time period of these regressions to the 11 months of 2022, omitting 2021 (Supp. Fig 21). With the exception of Romania (and to an extent Bulgaria and Spain), all pass-through coefficients are remarkably similar in each regression subsample (see Extended Data Fig. 6 for the main sample estimates). In the case of Romania and Bulgaria, one can speculate that there may be either significant changes in the capacity mix or manipulative bidding occurring during 2022, while in Spain the announcement of the so-called "Iberian exception" of natural gas pricing may play a role. Similarly, a robustness check is conducted for the sample of January 2021-January 2023, in which the results are also qualitatively similar (Supp. Fig. 22). A further detailed analysis of robustness checks of natural gas price-through during the energy crisis can be found in [19].

	BG	CZ	DE	DK	ES	FI	GR	HR	HU	IT	NL	PL	RO
NG Price (EUR/MWh)	1.251***	1.709***	1.717***	1.783***	0.586***	0.713***	1.436***	1.935***	1.896***	1.792***	1.862***	0.494***	1.819***
	(0.137)	(0.129)	(0.128)	(0.147)	(0.130)	(0.208)	(0.107)	(0.134)	(0.128)	(0.126)	(0.129)	(0.098)	(0.123)
IRE (GWh)	-94.697***	-78.342***	-6.180***	-40.326***	-5.225***	-51.336***	-28.427***	-39.218***	-16.508**	-6.516***	-25.789***	-18.968***	-41.489***
	(14.197)	(7.791)	(0.213)	(2.009)	(0.378)	(4.372)	(2.657)	(9.282)	(8.063)	(0.922)	(2.036)	(0.871)	(3.202)
Load (GWh)	96.062***	69.450***	12.793***	53.288**	6.237*	167.227***	28.011**	185.979***	178.898***	6.208***	49.104***	38.107***	-22.897**
	(31.518)	(16.082)	(1.618)	(23.451)	(3.313)	(27.577)	(14.091)	(59.241)	(32.371)	(2.062)	(5.723)	(5.212)	(10.091)
Load Sq. (GWh)	-2.719	-2.420**	-0.072***	-3.042	-0.008	-6.328***	-0.140	-7.942	-11.830***	0.018	-1.704***	-0.719***	5.902***
	(3.291)	(0.984)	(0.013)	(2.608)	(0.057)	(1.367)	(1.121)	(12.617)	(3.072)	(0.029)	(0.271)	(0.129)	(0.839)
Observations	7,680	7,680	7,680	7,680	7,680	7,680	7,680	7,680	7,680	7,680	7,680	7,680	7,680
R-squared	0.681	0.796	0.875	0.803	0.718	0.524	0.680	0.826	0.813	0.884	0.816	0.692	0.814
Month FE	YES	YES	YES	YES	YES	YES	YES	YES	YES	YES	YES	YES	YES
Hour FE	YES	YES	YES	YES	YES	YES	YES	YES	YES	YES	YES	YES	YES
Day of Week FE	YES	YES	YES	YES	YES	YES	YES	YES	YES	YES	YES	YES	YES

Clustered standard errors on day in parentheses. Sample: January 2022-November 2022

*** p<0.01, ** p<0.05, * p<0.1

Supplementary Tab. 26: Pass-through results for all countries across our main specification, but for a different period of January 2022-December 2022. Pass-through coefficients and standard errors (in parenthesis) displayed for every country. The natural gas price coefficient represents the impact of an increase in the price of natural gas (in EUR/MWh) on the wholesale electricity price (in EUR/MWh) for each country. The statistical test used is the two-sided t-test. * indicates significance at the 10% level, ** indicates significance at the 5% level, and *** indicates significance at the 1% level.

	(1) BG	(2) CZ	(3) DE	(4) DK	(5) ES	(6) FI	(7) HR	(8) HU	(9) IT	(10) NL	(11) PL	(12) RO
Natural Gas Price (EUR/GWh)	1.117***	1.866***	1.872***	1.711***	0.773***	1.028***	1.946***	1.987***	2.060***	1.856***	0.986***	1.300***
	(0.0415)	(0.0373)	(0.0354)	(0.0409)	(0.0452)	(0.0547)	(0.0349)	(0.0355)	(0.0302)	(0.0316)	(0.0310)	(0.0693)
Intermittent Renewables (GWh)	-75.65***	-59.31***	-4.404***	-30.61***	-5.886***	-32.58***	-16.95***	-11.34**	-5.261***	-18.86***	-12.58***	-19.40***
	(10.49)	(4.789)	(0.161)	(1.333)	(0.477)	(2.877)	(6.426)	(4.929)	(0.648)	(1.266)	(0.695)	(3.756)
Load (GWh)	161.7***	56.93***	9.540***	27.57*	16.44***	38.98*	176.6***	107.8***	5.976***	42.91***	11.32**	-50.46***
	(29.38)	(10.60)	(1.210)	(16.25)	(4.351)	(20.41)	(42.57)	(24.27)	(1.604)	(4.164)	(4.597)	(5.715)
Load Squared (GWh)	-5.382*	-1.684**	-0.0432***	1.321	-0.196***	-0.348	-11.35	-5.174**	0.00174	-1.356***	-0.103	8.362***
	(3.058)	(0.655)	(0.00996)	(1.809)	(0.0723)	(1.024)	(9.408)	(2.274)	(0.0225)	(0.189)	(0.117)	(0.641)
Observations	16,440	16,440	16,440	16,440	16,440	16,440	16,440	16,440	16,440	16,440	16,440	16,440
R-squared	0.690	0.833	0.869	0.801	0.520	0.535	0.864	0.856	0.908	0.842	0.698	0.513
Month FE	YES	YES	YES	YES	YES	YES	YES	YES	YES	YES	YES	YES
Hour FE	YES	YES	YES	YES	YES	YES	YES	YES	YES	YES	YES	YES
Day of Week FE	YES	YES	YES	YES	YES	YES	YES	YES	YES	YES	YES	YES

Clustered Robust standard errors on day in parentheses *** $p<0.01$, ** $p<0.05$, * $p<0.1$

Supplementary Tab. 27: Pass-through results for all countries during a sample period of January 2021-January 2023. Pass-through coefficients and standard errors (in parenthesis) displayed for every country. The natural gas price coefficient represents the impact of an increase in the price of natural gas (in EUR/MWh) on the wholesale electricity price (in EUR/MWh) for each country. The statistical test used is the two-sided t-test. * indicates significance at the 10% level, ** indicates significance at the 5% level, and *** indicates significance at the 1% level.

1.3.3 Relative Responsiveness Index

Here we provide additional information in regards to the relative responsiveness index. The index quantifies the relationship between coal responsiveness and the pass-through of gas prices to electricity prices, capturing how shifts in relative fuel costs affect the balance of coal and gas generation. It is calculated as the correlation between hourly marginal effects of coal generation with respect to the relative price and the hourly pass-through of gas prices to electricity. A more positive value (after negating the index) reflects a stronger substitutability between coal and gas, where increases in gas prices lead to significant coal generation adjustments, typically seen in systems with higher shares of coal and gas in the generation mix (Supp. Fig 1 & Supp. Fig 2). Conversely, a negative or near-zero RRI suggests weaker substitution, potentially due to structural factors like high shares of IRE or decarbonized energy (Supp. Fig 2 & Fig 4), which act as an alternative, or operational constraints that limit switching flexibility. By focusing on hourly dynamics, the index accounts for variations in market conditions (e.g., peak vs. off-peak hours) and provides a consistent framework to assess how the interplay of coal and natural gas generation responds to changing fuel prices.

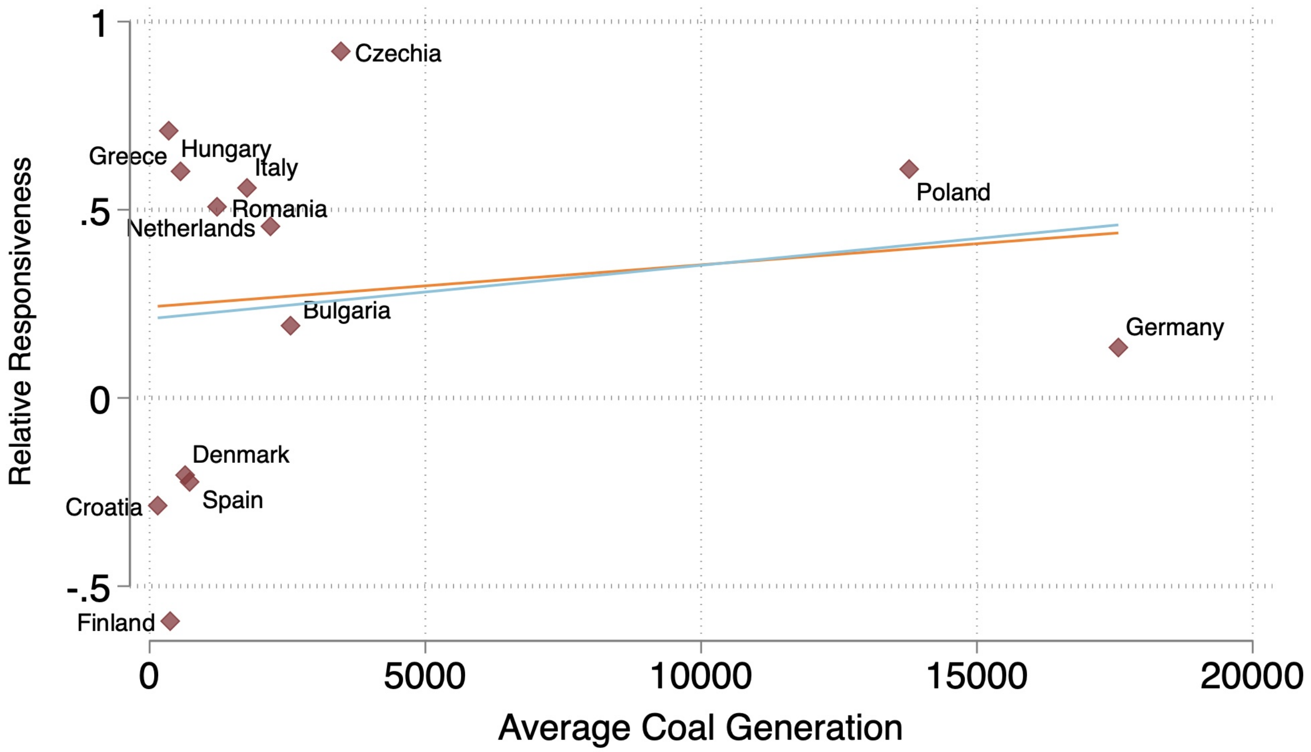

Supplementary Fig. 1: Relative Responsiveness relationship with absolute coal generation (MWh) across countries.Scatter plots include a fitted line of a linear regression estimated via OLS (blue line), and a fitted line estimated using the robust MM-estimator (orange line). The robust MM-estimator is preferred when the data has outliers and likely-influential but non-representative points [20].

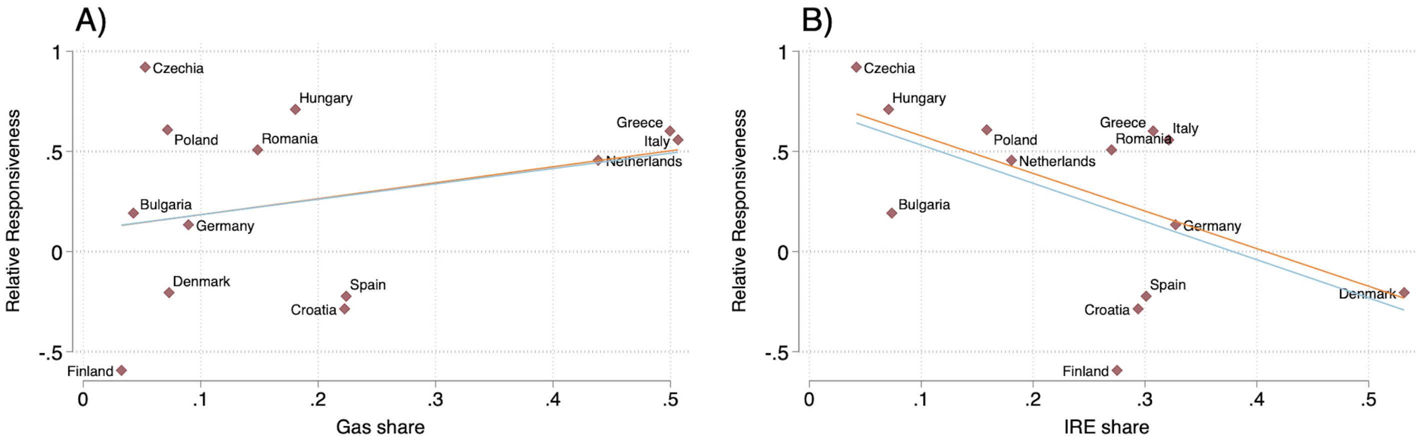

Supplementary Fig. 2: **A)** Relative Responsiveness relationship with natural gas energy generation across countries. **B)** Relative Responsiveness relationship with IRE generation across countries.

1.3.4 Policy analysis

Our carbon equivalent levy is calculated from the following equation:

$$\mathbb{E}_{2022}\left[\frac{P_{\text{Gas}}+(P_{\text{ETS}}+\Delta C)\,EF_{\text{Gas}}}{P_{\text{Coal}}+(P_{\text{ETS}}+\Delta C)\,EF_{\text{Coal}}}\right]=\mathbb{E}_{2022,\ \text{GasCap}}[\text{Relative Price}] \tag{1}$$

where ΔC is found to be 12.18 EUR/tonne. The right-hand side mean relative price of 2022 in this way is equated to the mean relative price of 2022 under the hypothetical scenario of the 180 EUR/MWh natural gas cap being imposed. *EF* refer to emissions factor of gas and coal, in which gas is 0.37 times as carbon intensive as coal. As mentioned in the methods, the process for finding this value was an iterative loop of incremental ΔC values by 0.01, until the absolute difference between both sides of the equation were closest as possible to zero.

Below we provided additional information on the two studied hypothetical policy effects during 2022.

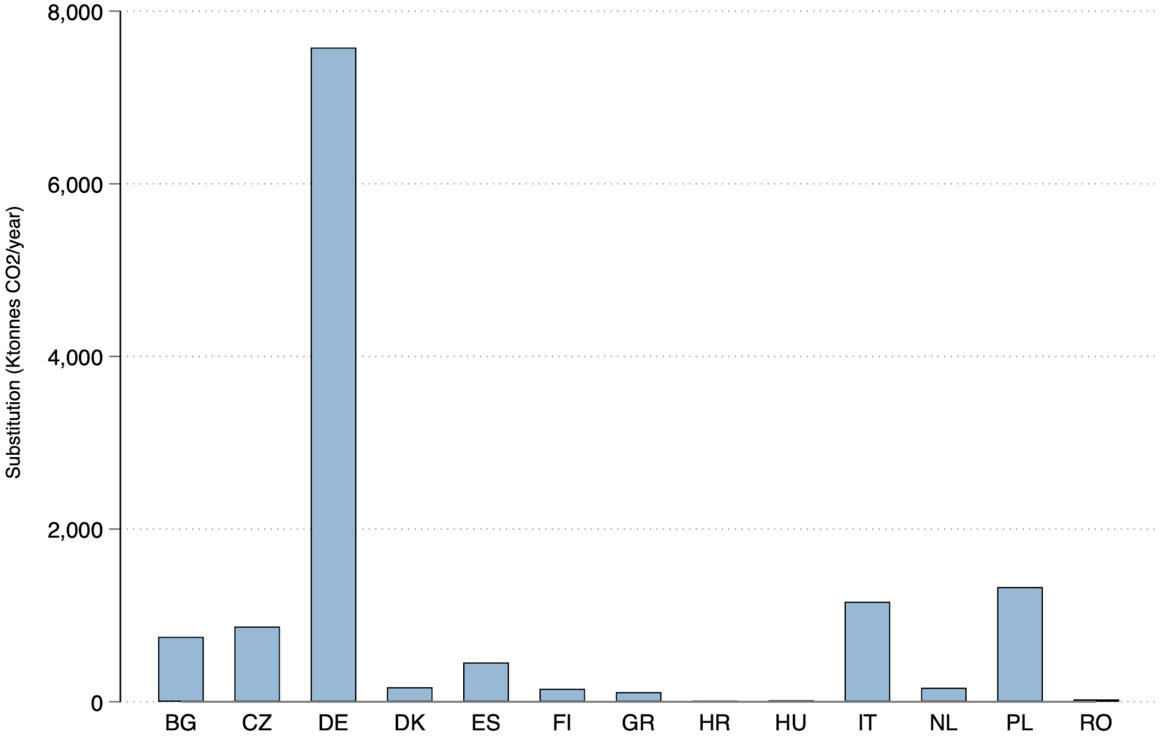

Supplementary Fig. 3: Substitution effect across countries under the 180 EUR/MWh natural gas price cap and equivalent 12.18 EUR/tonne carbon levy in 2022.

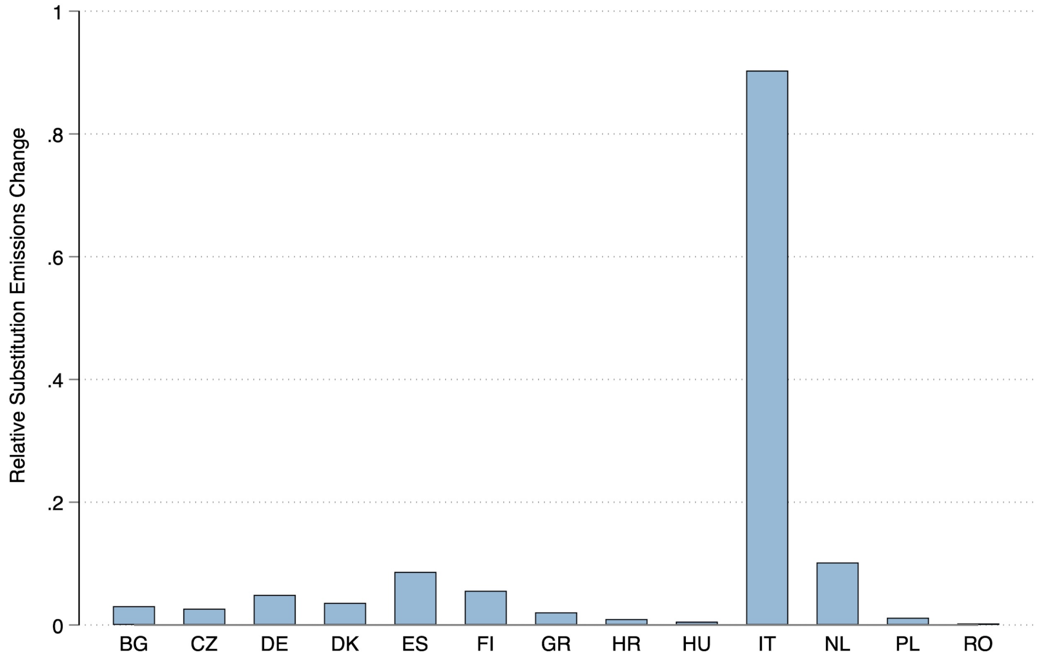

Supplementary Fig. 4: Substitution effect across countries under the 180 EUR/MWh natural gas price cap and equivalent 12.18 EUR/tonne carbon levy in 2022, in relation to baseline (January-April 2021 pre-crisis) emissions.

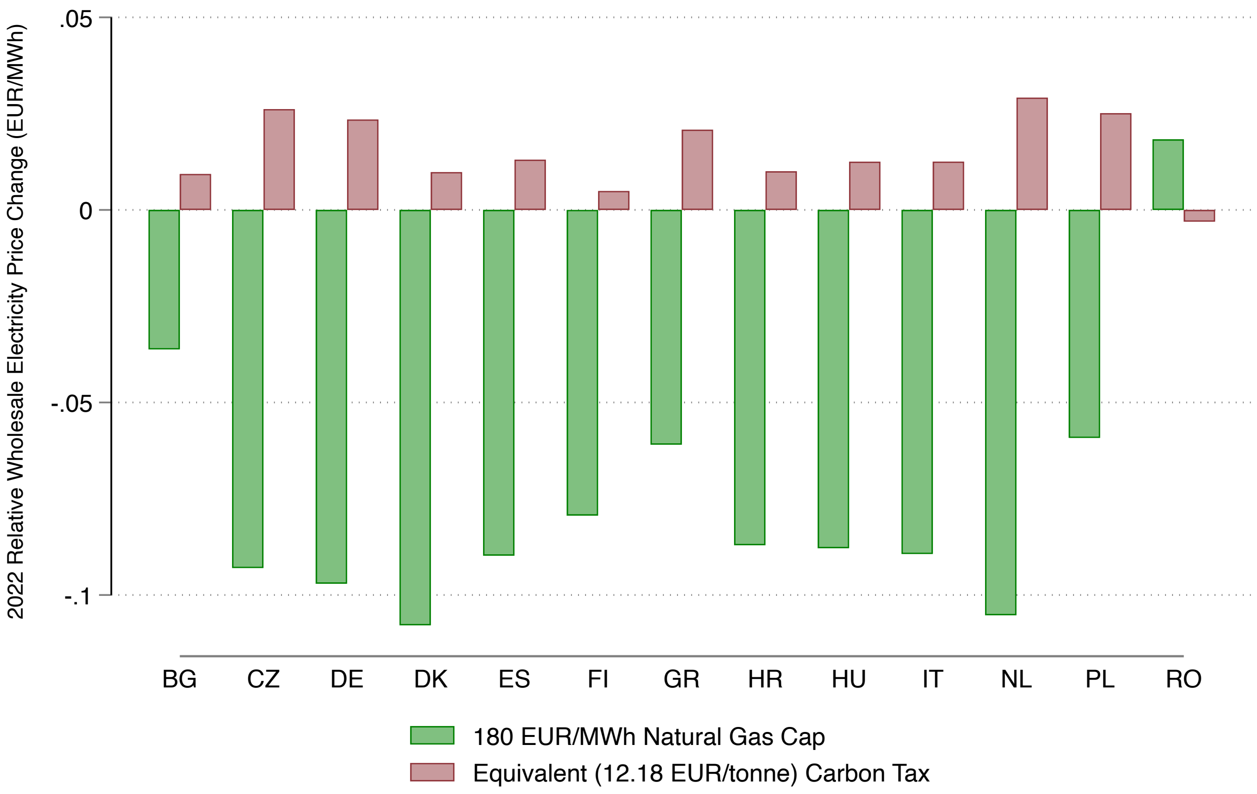

Supplementary Fig. 5: Wholesale electricity price change across countries under the two policies in 2022, in relation to baseline (January-April 2021 pre-crisis) prices.

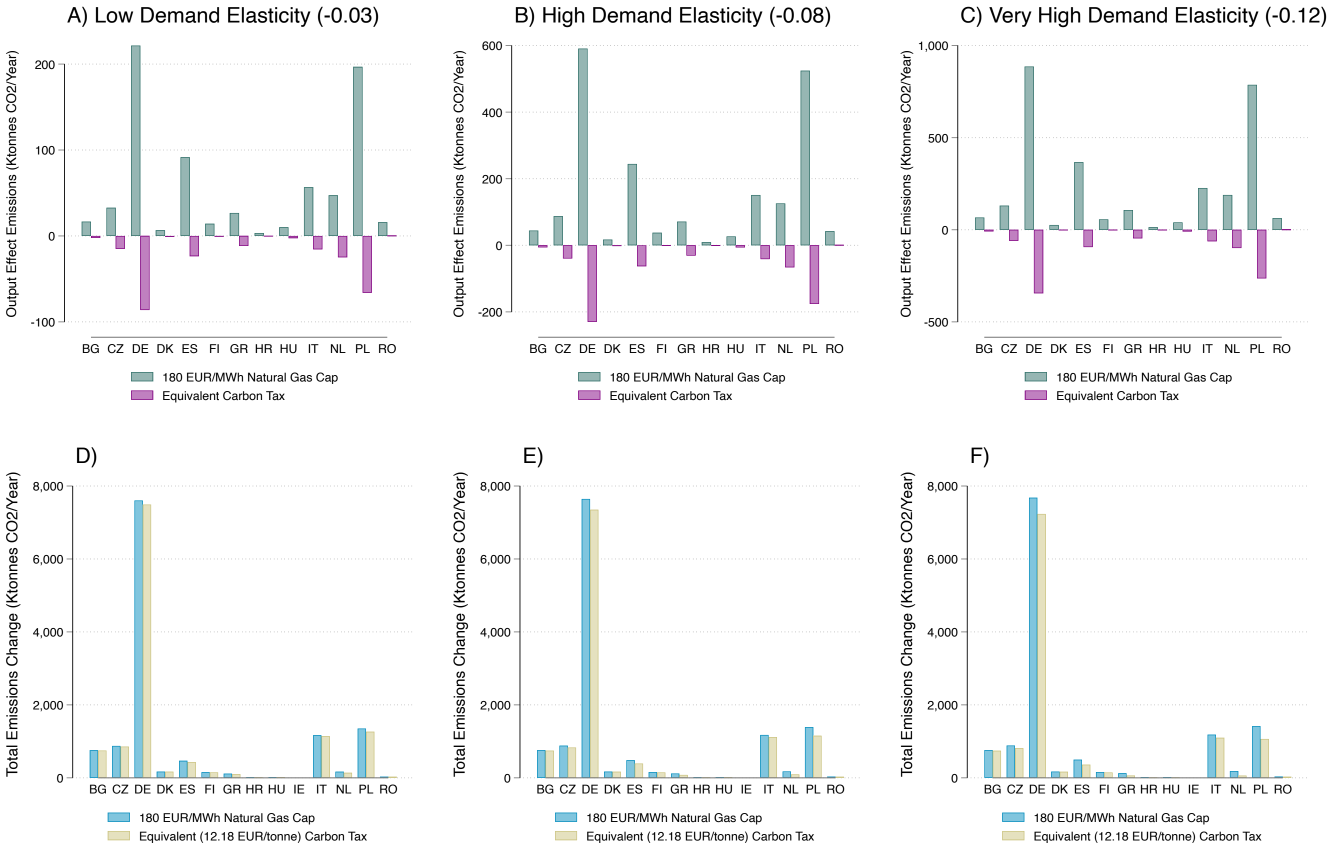

Supplementary Fig. 6: Output Effect emissions change under different elasticity of demand assumptions for natural gas cap and equivalent carbon tax policies. Since our output effect estimate relies on our electricity elasticity of demand assumption, we examine this effect under three different values of elasticity. As expected, these estimates vary considerably but always are less than the substitution effect. While results are evidently qualitatively similar between countries, the magnitude varies considerably depending on this assumption **A)** The low demand of elasticity refers to -0.03,in comparison to -0.06 which is used in the main Figure 5. The total emissions effect (addition of the substitution effect) is depicted in panel (D). **B)** The high demand of elasticity refers to -0.08, in comparison to 0.06 which is used in the main Figure 5. The total emissions effect (addition of the substitution effect) is depicted in Panel (E) **C)** The very high demand of elasticity refers to -0.12, in comparison to 0.15 which is used in the main Figure 5. The total emissions effect (addition of the substitution effect) is depicted in Panel (F).

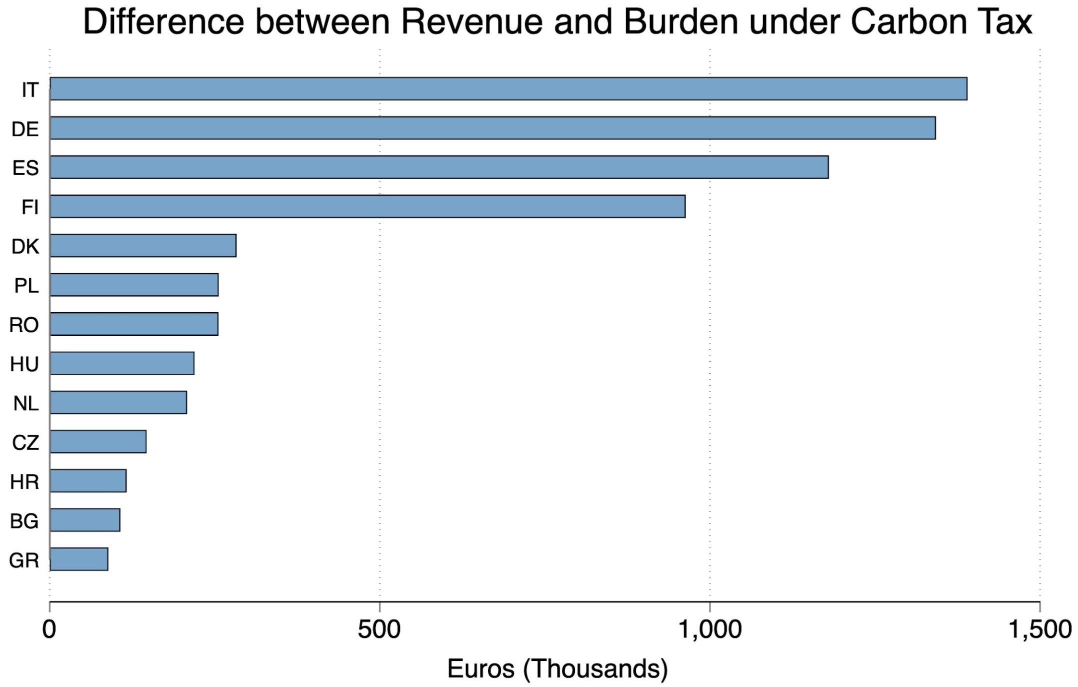

Supplementary Fig. 7: Difference between revenue generated from the emergency carbon levy and burden on consumers from higher electricity prices. This is calculated by subtracting the pink bars from the gray bars in Figure 5 Panel D

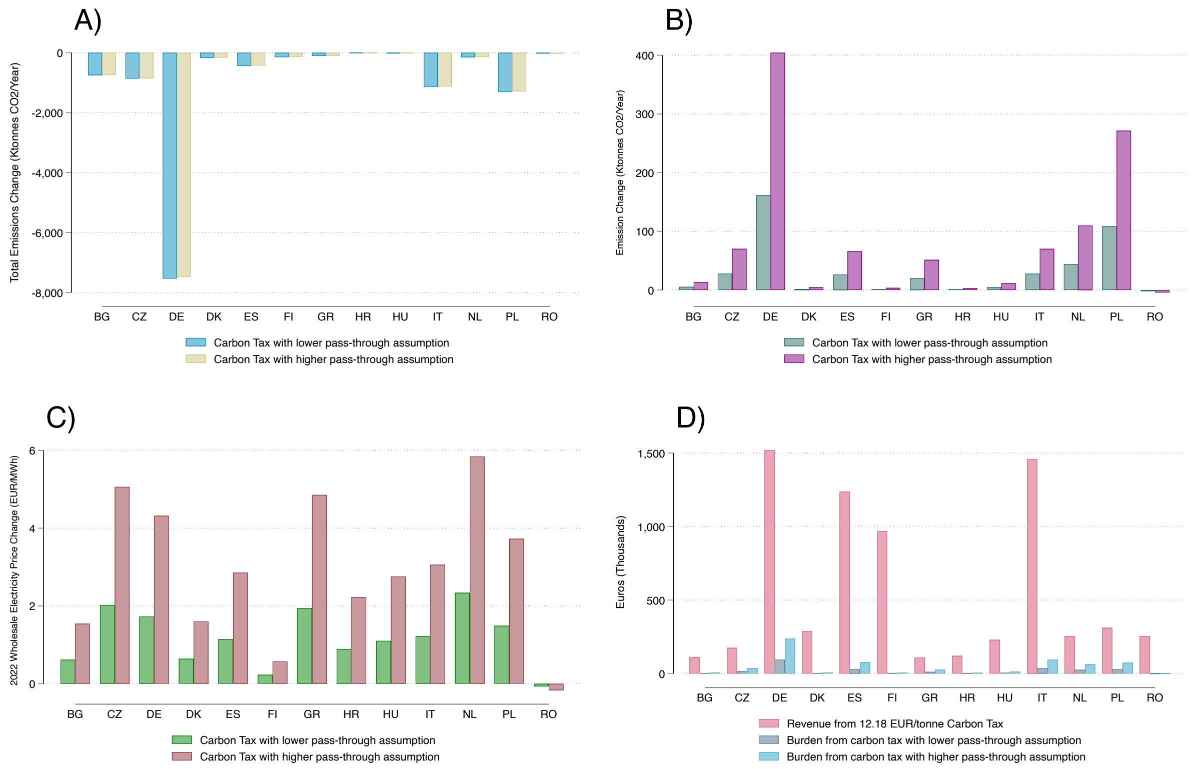

Supplementary Fig. 8: Policy counterfactual analysis: Different assumptions for the level of pass-through of the carbon price to electricity price during 2022. As our estimated pass-through reflects the pass-through of natural gas prices to wholesale electricity prices (which is the most common price-setter on the margin), our main analysis assumes that 0.37 of this pass-through is passed-through via a 1 euro/tonne increase in carbon price. We thus also estimate the increased electricity price from the carbon levy under a higher pass-through assumption (0.5) or a lower one (0.2). **A)** Comparison of wholesale electricity price change under each carbon tax assumption per country. **B)** Comparison of the change in revenue and financial burden and relief of each carbon tax assumption per country. The burden from the additional carbon pass-through and the revenues that could be used for consumer relief is shown. All values are normalized by average load per country, while the revenue and burden are adjusted with country specific emissions factors. **C)** Comparison of underlying output effect and substitution effect under each carbon tax assumption per country. The output effect per country is calculated using electricity price pass-through estimates and assumed elasticity of demand (-0.06), based on prior literature. **D)** Comparison of total emissions change under each carbon tax assumption per country, including the substitution and output effect.

2 Supplementary Tables

VARIABLES	(1) BG	(2) CZ	(3) DE	(4) DK	(5) ES	(6) FI	(7) GR	(8) HR	(9) HU	(10) IT	(11) IE	(12) NL	(13) PL	(14) RO
Relative Price	1.959***	1.279***	2.431***	1.277**	4.605***	3.269***	-0.244	0.455	0.092	4.324***	-2.109***	-1.883***	0.589***	0.135
	(0.244)	(0.280)	(0.307)	(0.541)	(0.592)	(0.460)	(0.855)	(0.401)	(0.270)	(0.603)	(0.759)	(0.642)	(0.078)	(0.194)
Intermittent Renewables (GWh)	-0.150***	-0.055***	-0.024***	-0.157***	-0.025***	-0.063***	-0.064***	-0.066***	0.059**	-0.040***	-0.335***	-0.110***	-0.054***	-0.055***
	(0.019)	(0.017)	(0.001)	(0.010)	(0.004)	(0.018)	(0.024)	(0.023)	(0.026)	(0.005)	(0.016)	(0.012)	(0.001)	(0.006)
Load (GWh)	0.311***	0.123***	0.075***	0.086	0.047	0.696***	0.455***	0.248**	0.168**	0.043**	0.083*	0.265***	0.135***	0.430***
	(0.045)	(0.044)	(0.008)	(0.161)	(0.035)	(0.122)	(0.087)	(0.118)	(0.077)	(0.019)	(0.050)	(0.023)	(0.009)	(0.044)
Load Squared (GWh)	-0.019***	-0.001	-0.000***	0.014	-0.001	-0.021***	-0.011*	-0.036	-0.006	-0.000	0.013	-0.010***	-0.002***	-0.023***
	(0.004)	(0.002)	(0.000)	(0.016)	(0.001)	(0.005)	(0.006)	(0.023)	(0.007)	(0.000)	(0.010)	(0.001)	(0.000)	(0.003)
Observations	10,223	10,224	10,224	10,223	10,193	8,882	10,213	7,981	10,210	10,224	9,974	10,206	10,224	10,173
R-squared	0.896	0.782	0.809	0.555	0.547	0.825	0.390	0.209	0.143	0.532	0.630	0.732	0.911	0.492
Month FE	YES	YES	YES	YES	YES	YES	YES	YES	YES	YES	YES	YES	YES	YES
Hour FE	YES	YES	YES	YES	YES	YES	YES	YES	YES	YES	YES	YES	YES	YES
Day of Week FE	YES	YES	YES	YES	YES	YES	YES	YES	YES	YES	YES	YES	YES	YES
**Marginal Effect**	**0.259*** **	**0.224*** **	**0.411*** **	**0.302*** **	**0.705*** **	**0.463*** **	**0.179*** **	**0.0836***	**0.0454**	**0.747*** **	**0.0295**	**0.0868**	**0.103*** **	**0.0206**
SE	Day Clustered	Day Clustered	Day Clustered	Day Clustered	Day Clustered	Day Clustered	Day Clustered	Day Clustered	Day Clustered	Day Clustered	Day Clustered	Day Clustered	Day Clustered	Day Clustered

Supplementary Tab. 28: Estimates of main regression results of responsiveness of log-transformed coal generation factor to relative price (natural gas price/coal price). The marginal effect, which is the estimate of interest, is derived through the delta method from the relative price coefficients, using the mean value of relative price during our sample period. The statistical test used is the two-sided t-test. * indicates significance at the 10% level, ** indicates significance at the 5% level, and *** indicates significance at the 1% level.

VARIABLES	BG	CZ	DE	DK	ES	FI	GR	HR	HU	IT	NL	PL	RO
Natural Gas Price	0.540***	1.621***	1.608***	1.584***	1.770***	0.834***	1.280***	1.744***	1.741***	1.964***	1.900***	0.792***	-0.090***
	(0.047)	(0.075)	(0.062)	(0.067)	(0.065)	(0.075)	(0.108)	(0.068)	(0.073)	(0.072)	(0.052)	(0.068)	(0.029)
Intermittent Renewables (GWh)	-43.011***	-54.841***	-4.407***	-28.461***	-4.745***	-20.316***	-15.996***	-15.998**	-17.658***	-5.120***	-17.302***	-11.296***	-4.824***
	(5.699)	(4.808)	(0.136)	(1.309)	(0.345)	(2.137)	(1.764)	(6.393)	(5.669)	(0.547)	(0.925)	(0.672)	(0.645)
Load (GWh)	-29.351*	-54.756***	-0.341	-108.089***	6.098*	-153.195***	7.571*	-34.895	-95.075***	4.113***	25.851***	-20.057***	-12.386***
	(16.339)	(9.468)	(1.004)	(19.643)	(3.457)	(23.157)	(4.516)	(33.422)	(18.464)	(1.195)	(4.664)	(5.123)	(1.376)
Observations	10,224	10,224	10,224	10,224	10,224	10,224	10,224	10,224	10,224	10,224	10,224	10,224	10,224
R-squared	0.778	0.780	0.873	0.753	0.877	0.638	0.817	0.842	0.831	0.902	0.829	0.721	0.603
Month FE	YES	YES	YES	YES	YES	YES	YES	YES	YES	YES	YES	YES	YES
Hour FE	YES	YES	YES	YES	YES	YES	YES	YES	YES	YES	YES	YES	YES
Day of Week FE	YES	YES	YES	YES	YES	YES	YES	YES	YES	YES	YES	YES	YES

Supplementary Tab. 29: Estimates of the main regression results of natural gas price pass-through to wholesale electricity prices for all countries during our main sample period. The Natural Gas price coefficients represent the impact of an increase in the price of natural gas (in EUR/MWh) on the wholesale electricity price (in EUR/MWh) for each country. The statistical test used is a two-sided t-test. * indicates significance at the 10% level, ** indicates significance at the 5% level, and *** indicates significance at the 1% level. Standard errors are clustered at a daily level. See Supplementary Discussion 1.2 for missing country exception.

3 Supplementary Figures

Country Selection

EU Countries	Code	Present?	Reason for Absence
Austria	AT		No coal
Belgium	BE		No coal
Bulgaria	BG	y	
Croatia	HR	y	
Republic of Cyprus	CY		No coal
Czech Republic	CZ	y	
Denmark	DK	y	
Estonia	EE		Small quantity of coal-derived gas
Spain	ES	y	
Finland	FI	y	
France	FR		Small quantity of coal, mostly nuclear
Germany	DE	y	
Greece	GR	y	
Hungary	HU	y	
Ireland	IE	y	
Italy	IT	y	
Latvia	LV		No coal
Lithuania	LT		No coal
Luxembourg	LU		No coal
Malta	MT		No data
Netherlands	NL	y	
Poland	PL	y	
Portugal	PT		Small quantity of coal
Romania	RO	y	
Slovakia	SK		Small quantity of coal, mostly nuclear
Slovenia	SI		Small quantity of gas
Sweden	SE		No coal

Created with Datawrapper

Supplementary Fig. 9: Explanation of country selection for our analysis. Requirements include the significant presence of coal and natural gas generation and evidently adequate non-missing data.

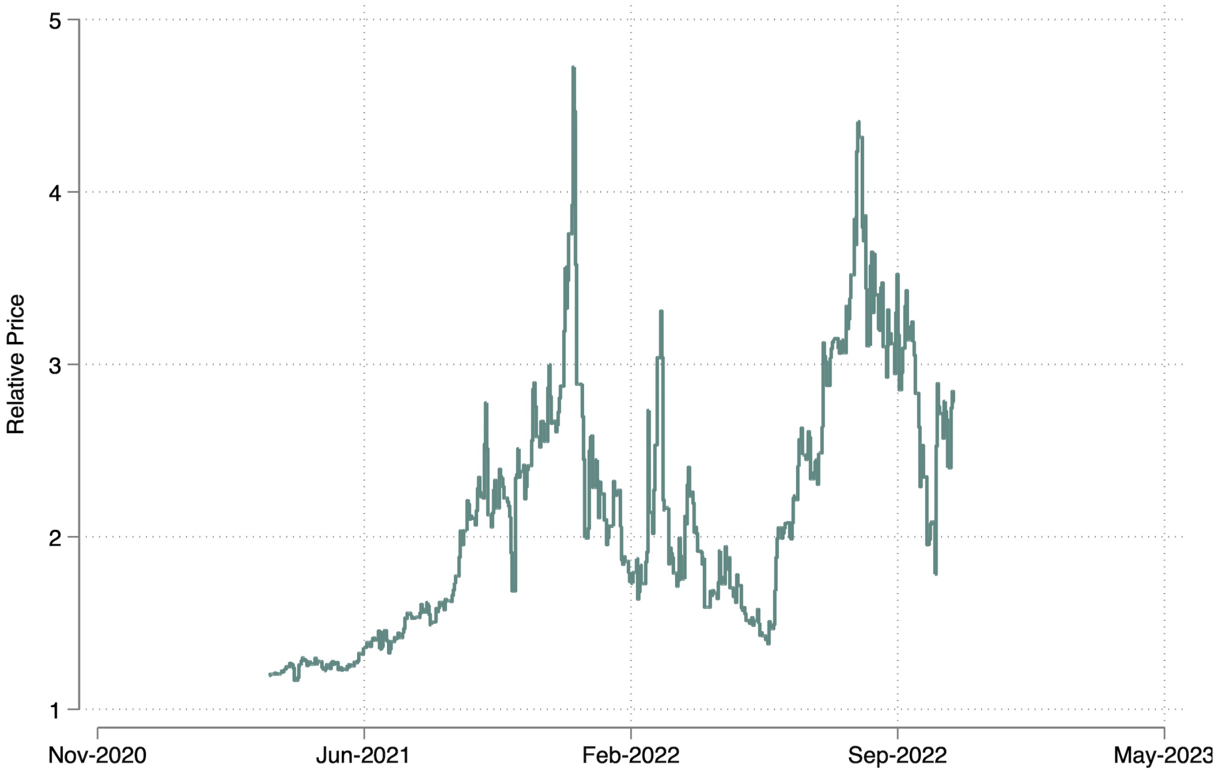

Supplementary Fig. 10: Timeseries of the EU relative (natural gas/coal) price from January 2021 to December 2022, inclusive of carbon prices.

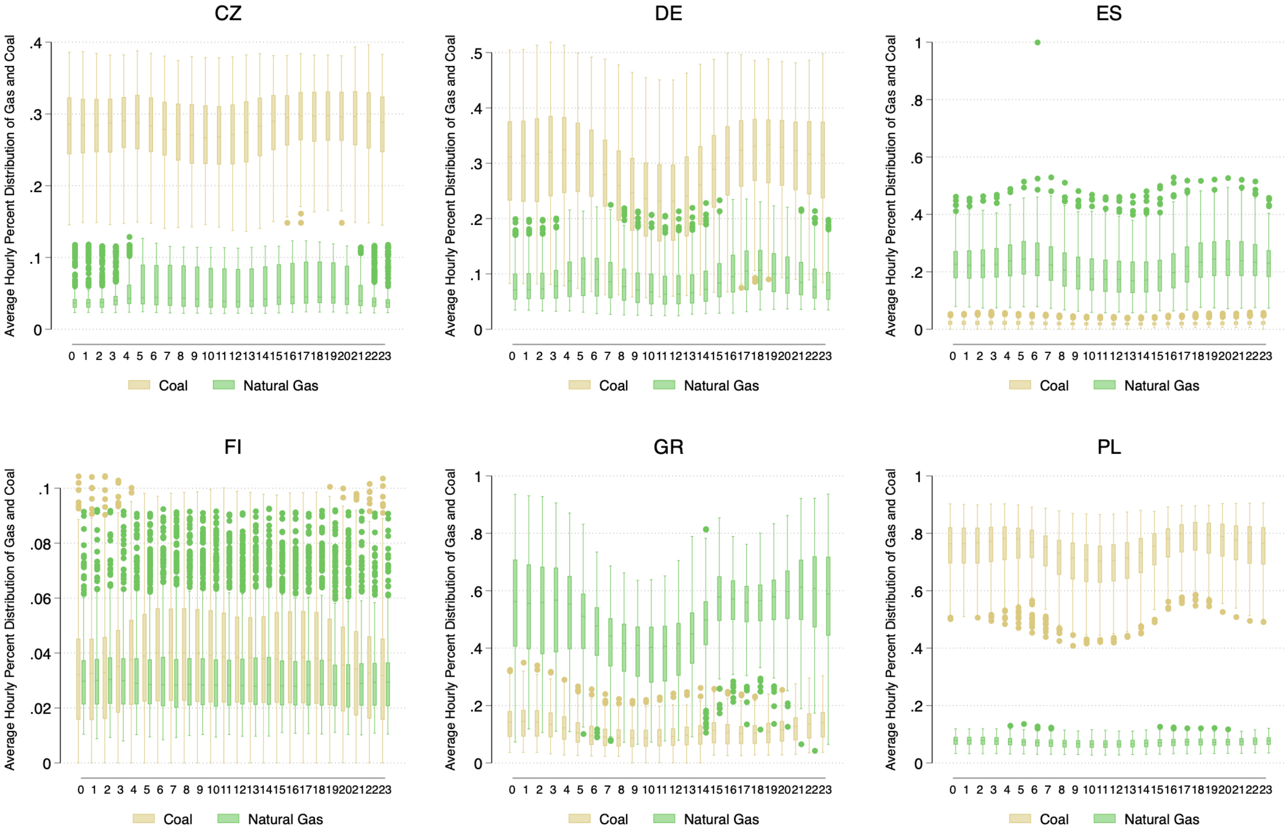

Supplementary Fig. 11: The hourly distribution of coal and natural gas energy generation (as a share) for our subset of countries, with vertical box plots capturing the ±1.5∗IQR (the interquartile range) with bounds at the 25th percentile (Q1) and 75th percentile (Q3). The center line of the box represents the median (50th percentile). Whiskers extend to the smallest and largest values within 1.5 × IQR of the lower and upper quartiles, respectively, while dots correspond to the outliers outside this range.

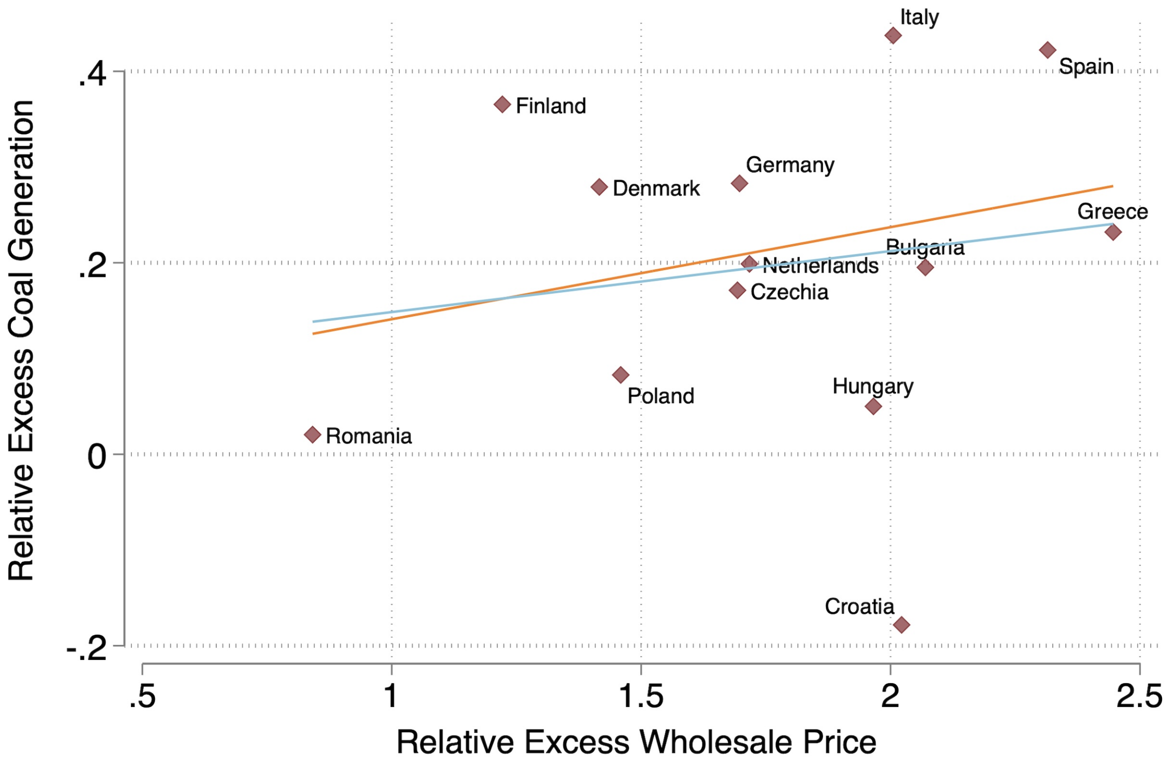

Supplementary Fig. 12: Relationship of excess coal with excess wholesale electricity price across countries, both in relative terms (using the January-April 2021 pre-crisis average as a baseline).

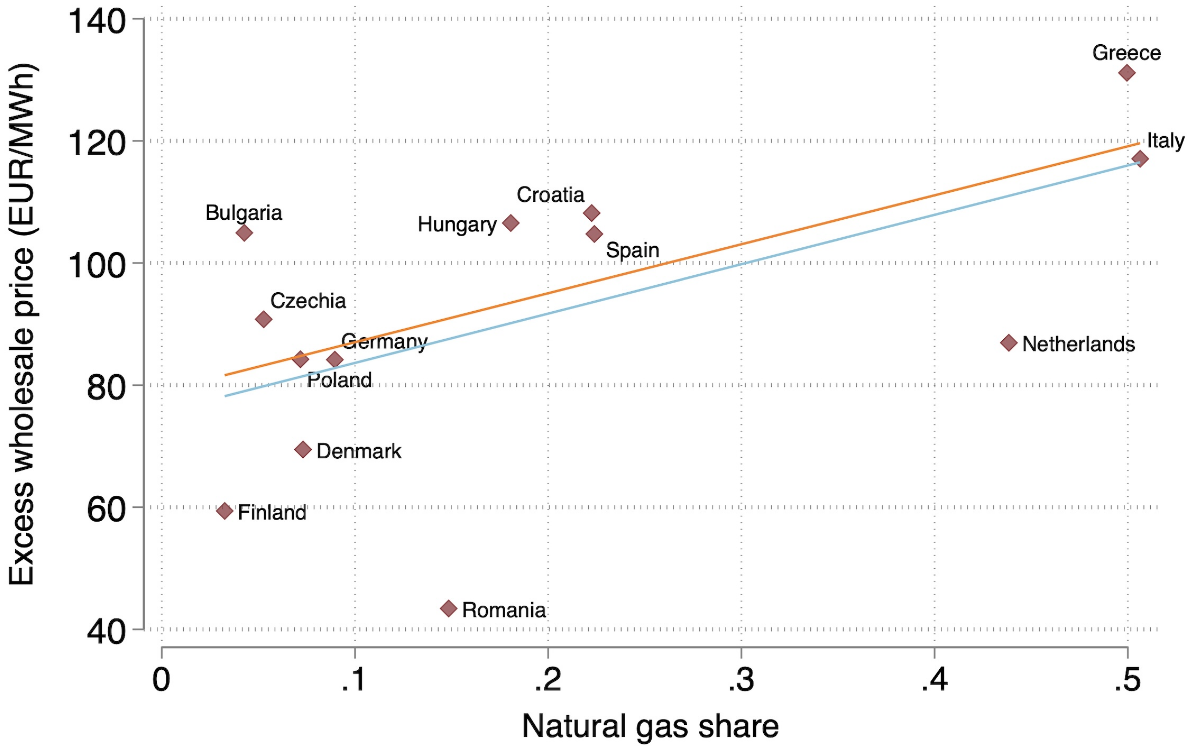

Supplementary Fig. 13: Relationship of excess wholesale electricity price across countries, in relationship to natural gas share.

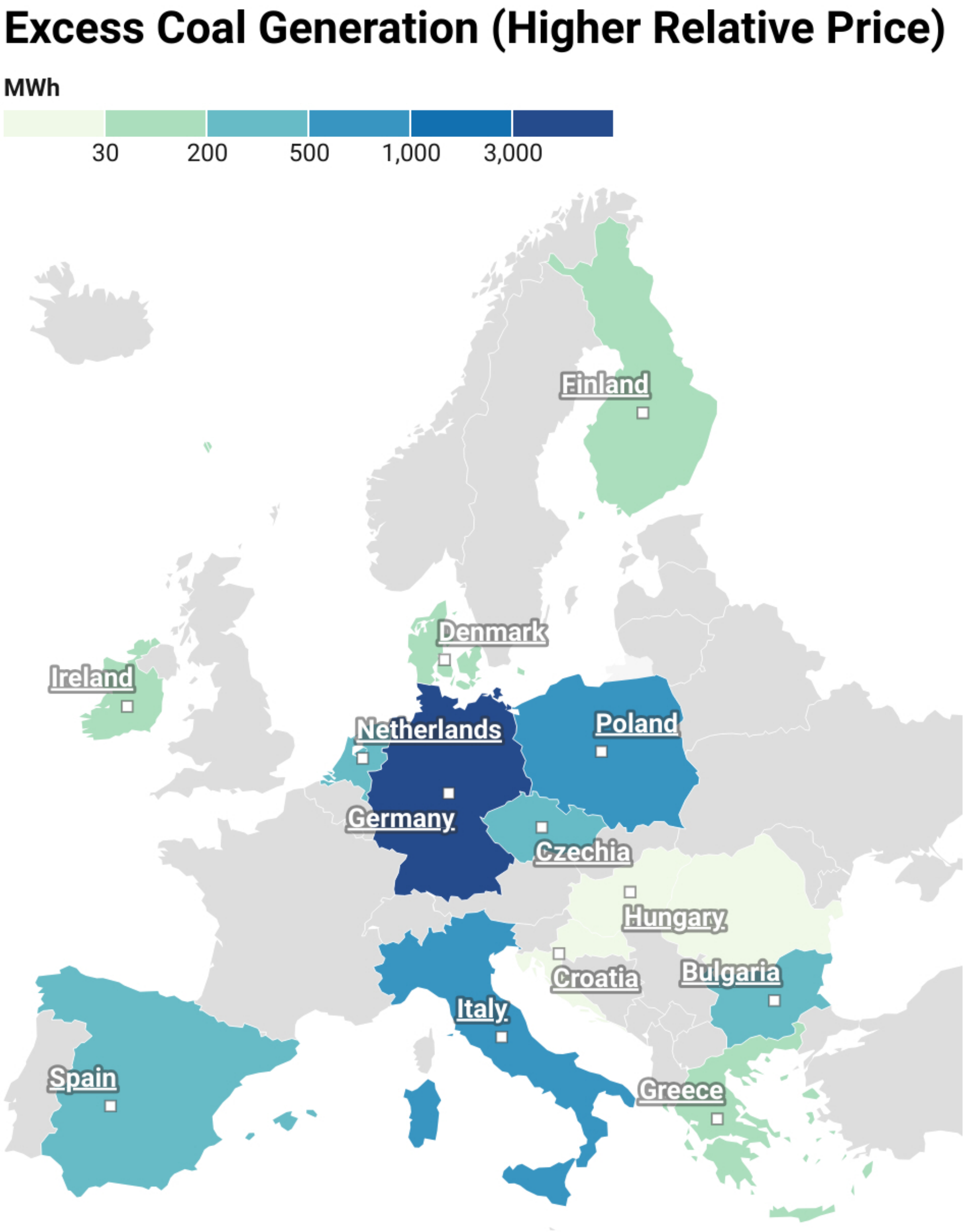

Supplementary Fig. 14: Excess coal and lignite generation during April 1 2021 - May 30 2022, using a higher counterfactual relative price (compared to the actual January-April 2021) during the pre-crisis period. Excess coal refers to the additional amount of coal generated in surplus of the predicted coal under "non-crisis price environment." This is calculated by subtracting the estimated amount of coal generated under counterfactual scenario where relative gas prices are equivalent to the pre-crisis period January - March 2021 (the 3rd quartile) – from the actual observed coal generation (panel D), as discussed in Methods. The map was created by the authors using data visualization tools provided by Datawrapper.

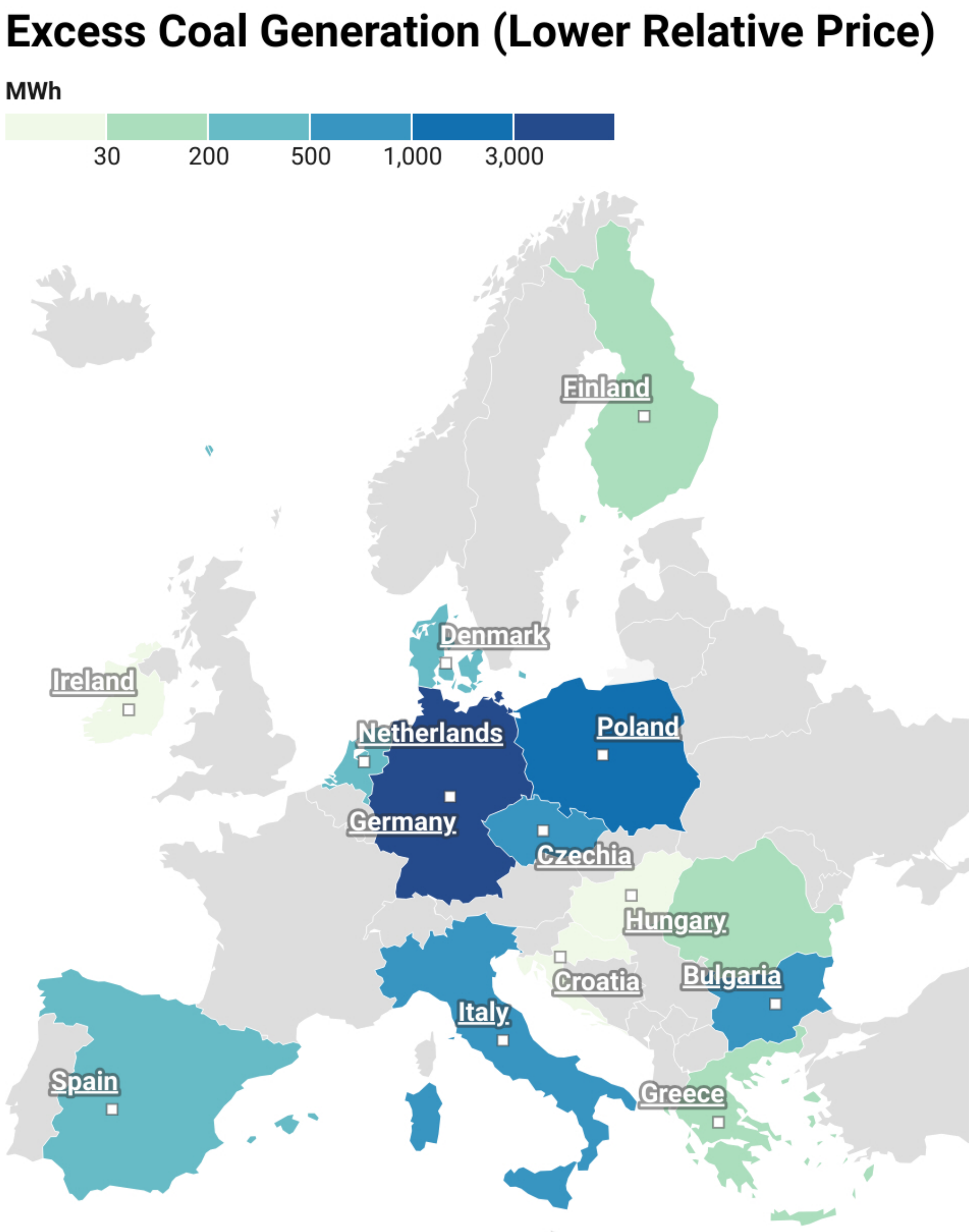

Supplementary Fig. 15: Excess coal and lignite generation during April 1 2021 - May 30 2022, using a lower counterfactual relative price (compared to the actual January-April 2021) during the pre-crisis period. Excess coal refers to the additional amount of coal generated in surplus of the predicted coal under "non-crisis price environment." This is calculated by subtracting the estimated amount of coal generated under counterfactual scenario where relative gas prices are equivalent to the pre-crisis period January - March 2021 (the 1st quartile) – from the actual observed coal generation (panel D), as discussed in Methods. The map was created by the authors using data visualization tools provided by Datawrapper.

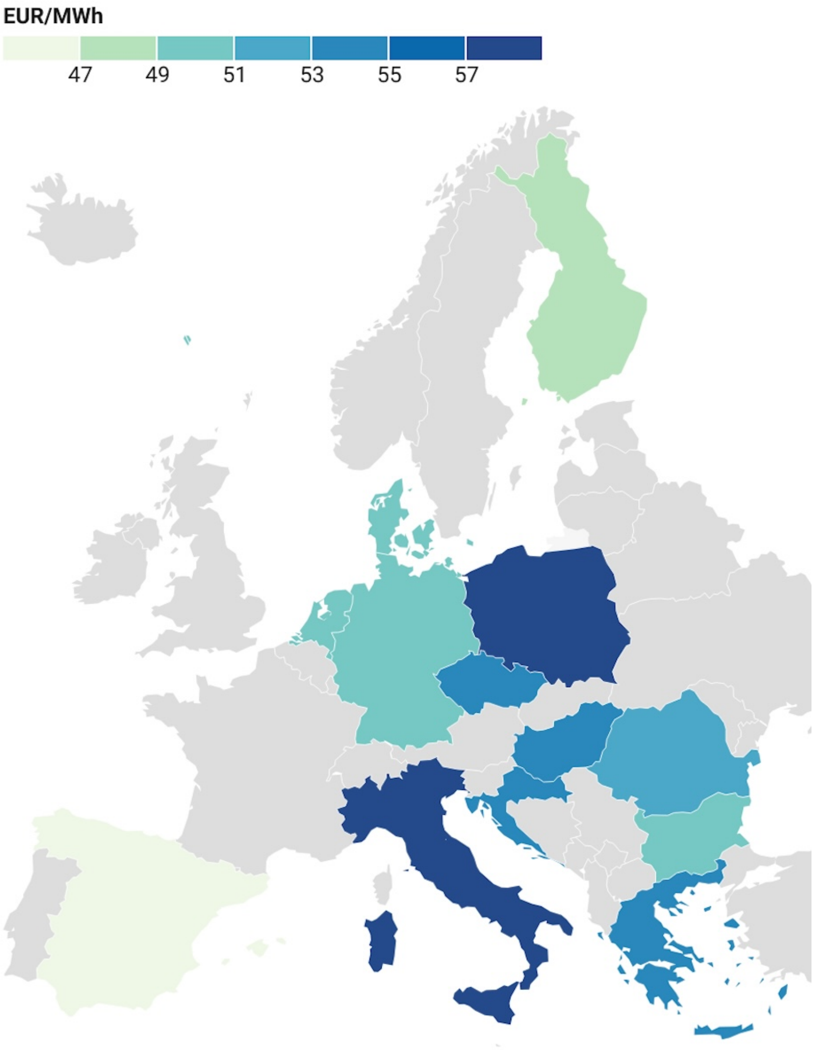

Supplementary Fig. 16: Pre-crisis wholesale electricity prices (January-April 2021) across sample countries. The map was created by the authors using data visualization tools provided by Datawrapper.

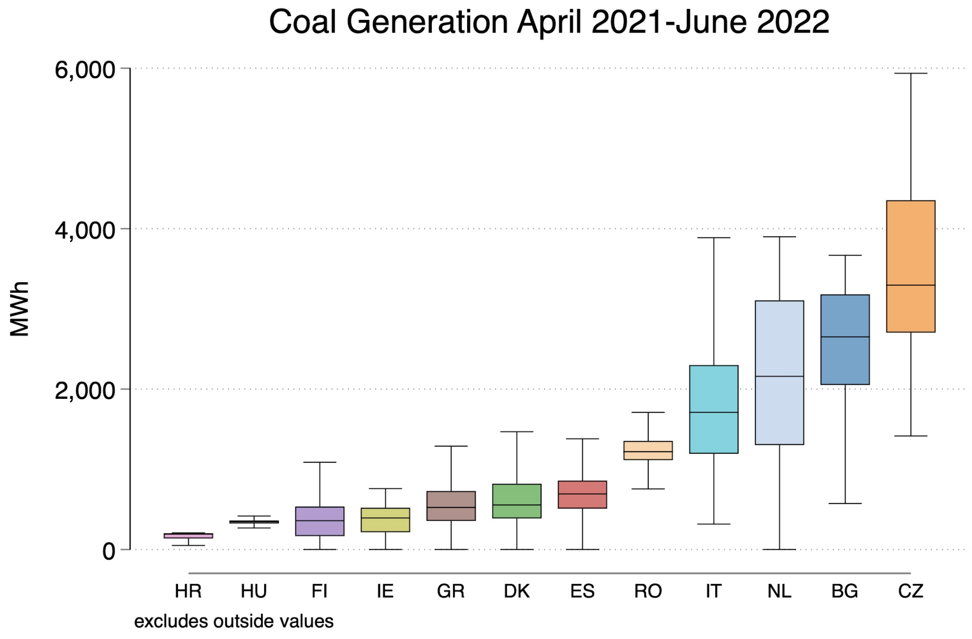

Supplementary Fig. 17: Distribution of coal Generation during sample period, excluding Germany and Poland, with vertical box plots capturing the $\pm 1.5 * \text{IQR}$ (the interquartile range) with bounds at the 25th percentile (Q1) and 75th percentile (Q3). The center line of the box represents the median (50th percentile). Whiskers extend to the smallest and largest values within $1.5 \times \text{IQR}$ of the lower and upper quartiles, respectively, while dots correspond to the outliers outside this range.

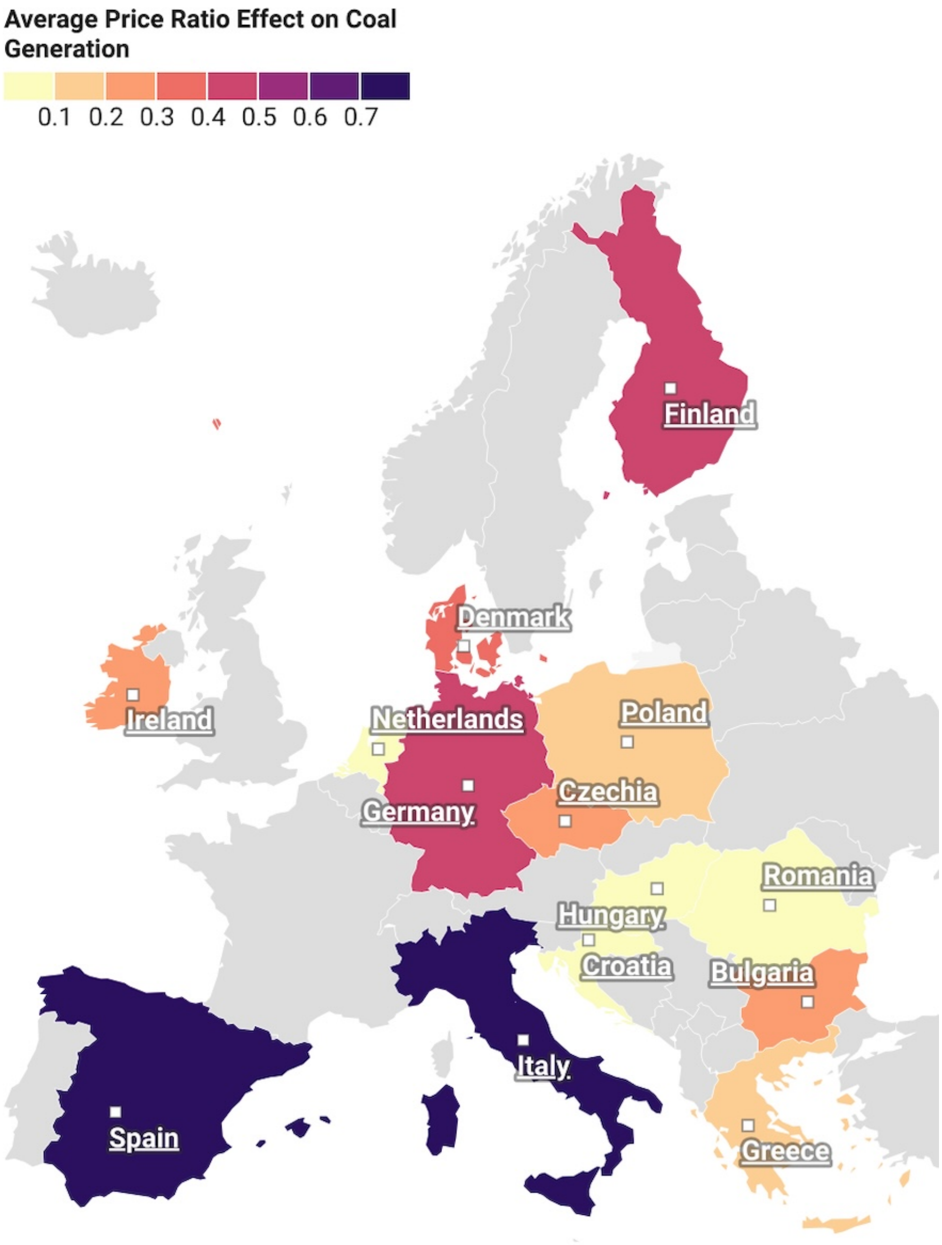

Supplementary Fig. 18: Estimates of coal responsiveness estimates under main specification during main sample period from April 2021-June 2022. The map was created by the authors using data visualization tools provided by Datawrapper.

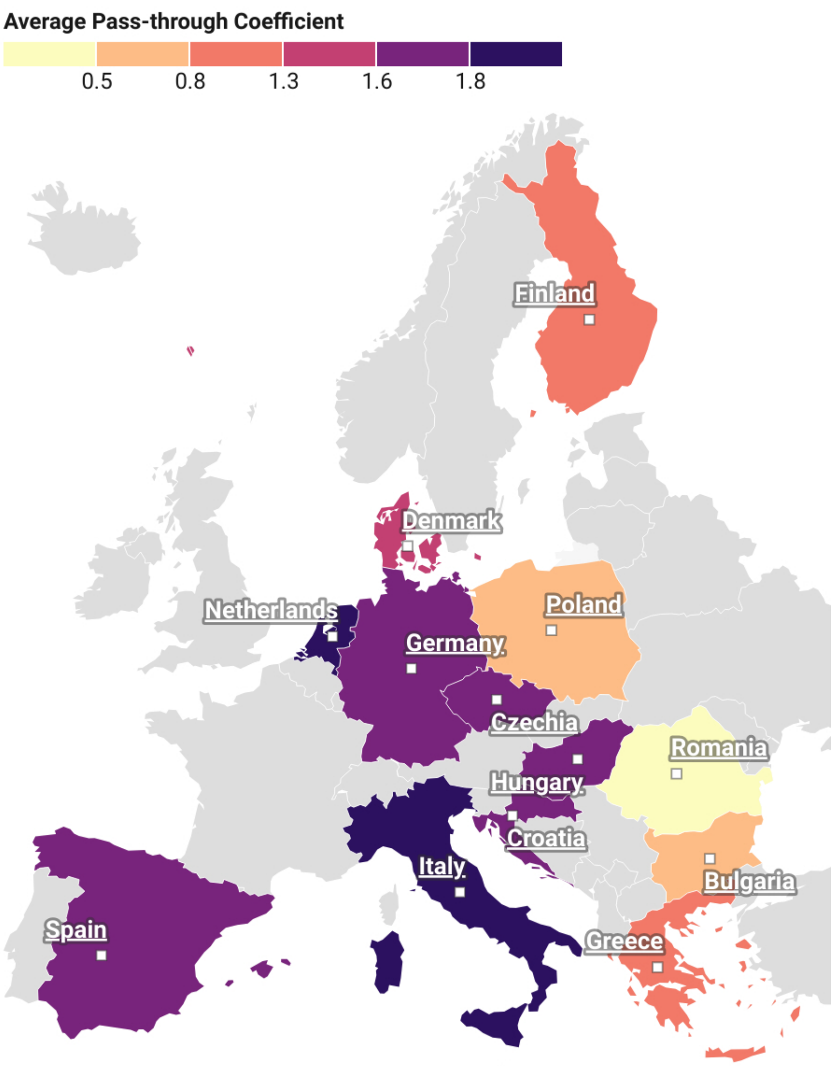

Supplementary Fig. 19: Estimates from pass-through regression across countries of natural gas TTF price to wholesale electricity price during our main sample period from April 2021-June 2022. The map was created by the authors using data visualization tools provided by Datawrapper.

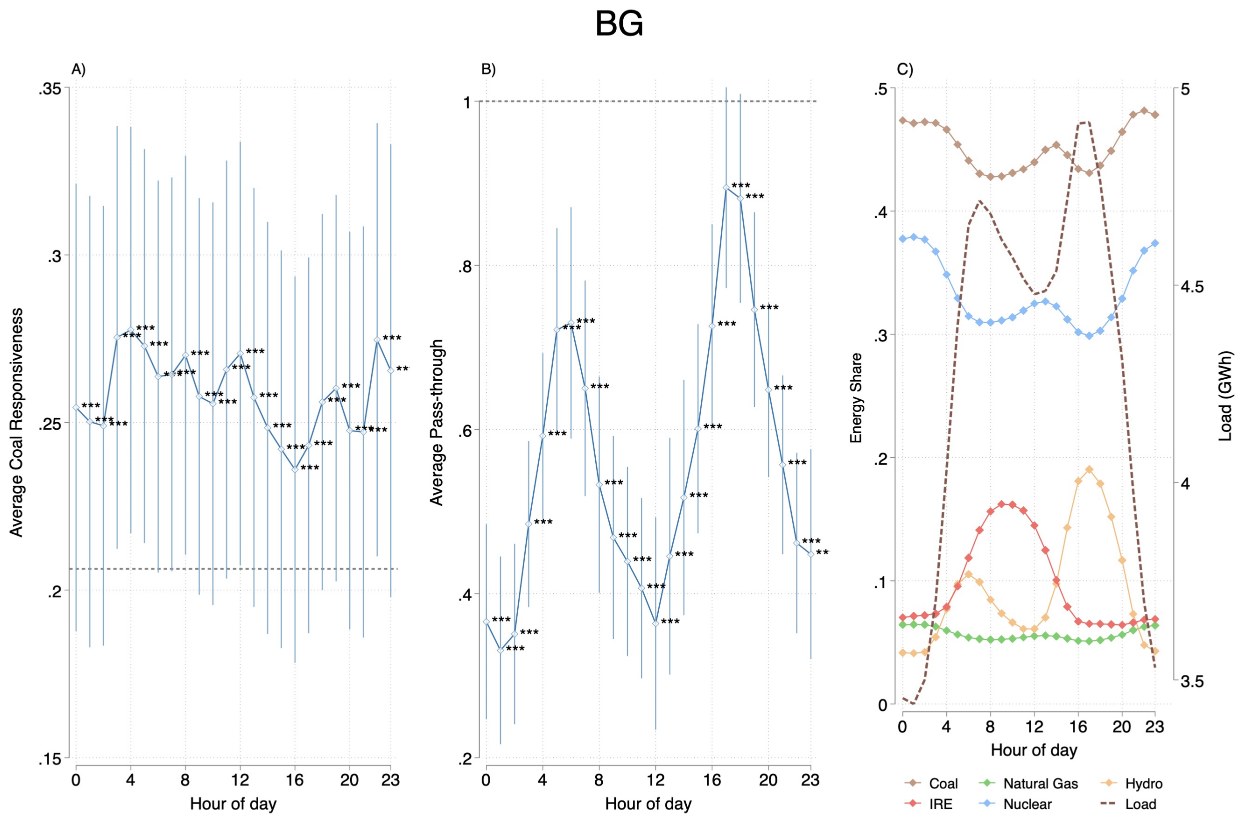

Supplementary Fig. 20: A) Estimated hourly coal responsiveness coefficients; B) estimated pass-through coefficients by hour; C) the average share of different generation technologies used by hour for Bulgaria, using our main specifications for each country (Supplementary Tables 28 and 29) while including an hourly interaction term with our regressor of interest. Dots in panels A) and B) represent the estimated hourly coal responsiveness or pass-through coefficients respectively, while the vertical lines capture 95% confidence intervals (± 1.96 times the standard error of each point estimate). The statistical test used is a two-sided t-test. The coefficients that are statistically distinct from either 0 or 1 at the 1% or 5% significance level are indicated with three stars or two stars, respectively. In the right panel, "intermittent renewable" includes solar, wind, and hydro-run-of-river. The dashed line displays the average hourly load.

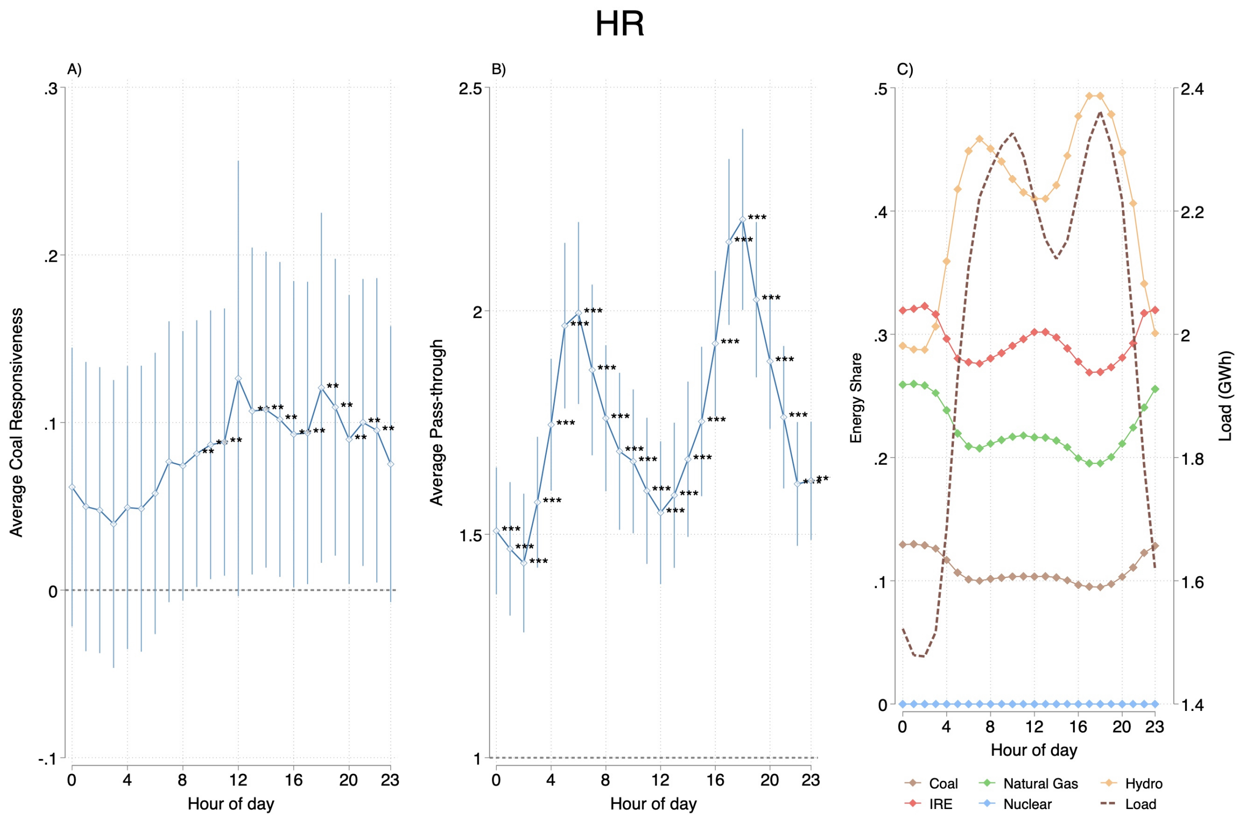

Supplementary Fig. 21: EA) Estimated hourly coal responsiveness coefficients; B) estimated pass-through coefficients by hour; C) the average share of different generation technologies used by hour for Croatia, using our main specifications for each country (Supplementary Tables 28 and 29) while including an hourly interaction term with our regressor of interest. Dots in panels A) and B) represent the estimated hourly coal responsiveness or pass-through coefficients respectively, while the vertical lines capture 95% confidence intervals (± 1.96 times the standard error of each point estimate). The statistical test used is a two-sided t-test. The coefficients that are statistically distinct from either 0 or 1 at the 1% or 5% significance level are indicated with three stars or two stars, respectively. In the right panel, "intermittent renewable" includes solar, wind, and hydro-run-of-river. The dashed line displays the average hourly load.

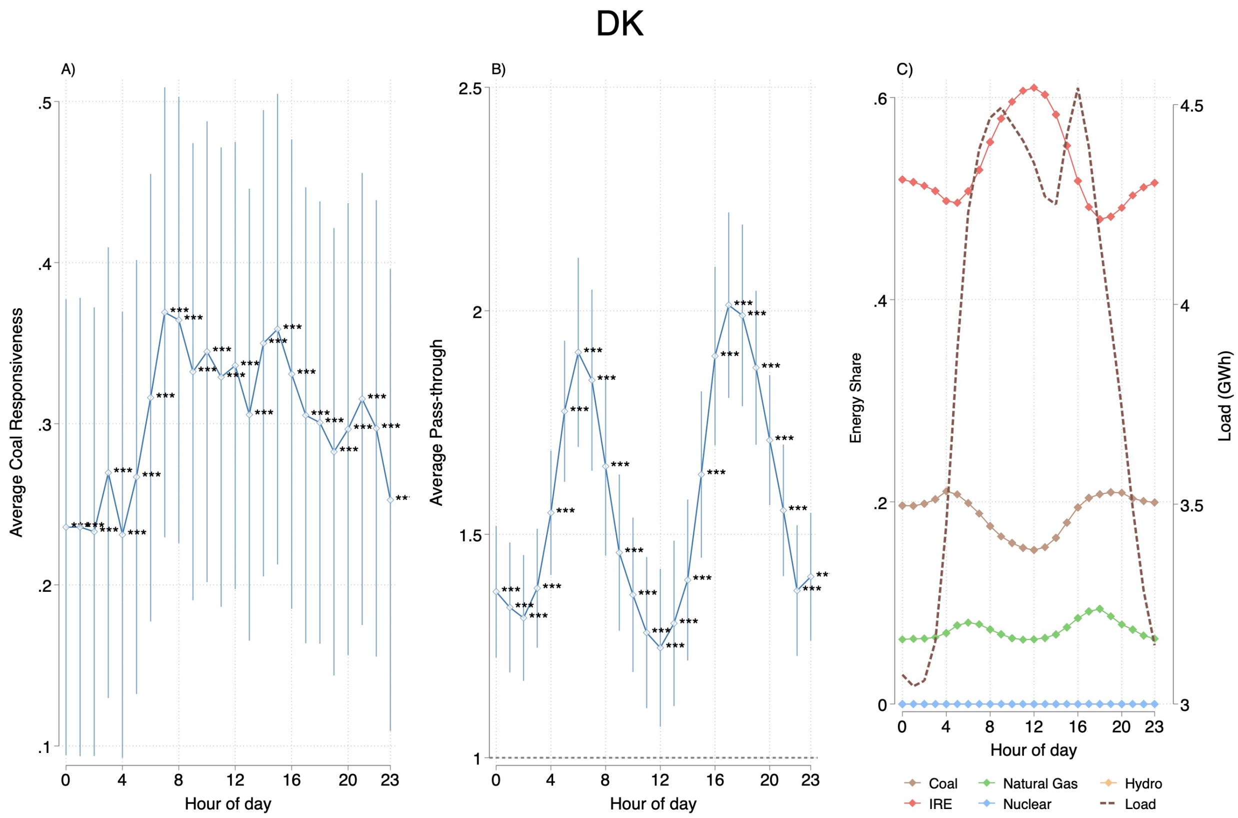

Supplementary Fig. 22: A) Estimated hourly coal responsiveness coefficients; B) estimated pass-through coefficients by hour; C) the average share of different generation technologies used by hour for Denmark, using our main specifications for each country (Supplementary Tables 28 and 29) while including an hourly interaction term with our regressor of interest. Dots in panels A) and B) represent the estimated hourly coal responsiveness or pass-through coefficients respectively, while the vertical lines capture 95% confidence intervals (± 1.96 times the standard error of each point estimate). The statistical test used is a two-sided t-test. The coefficients that are statistically distinct from either 0 or 1 at the 1% or 5% significance level are indicated with three stars or two stars, respectively. In the right panel, "intermittent renewable" includes solar, wind, and hydro-run-of-river. The dashed line displays the average hourly load.

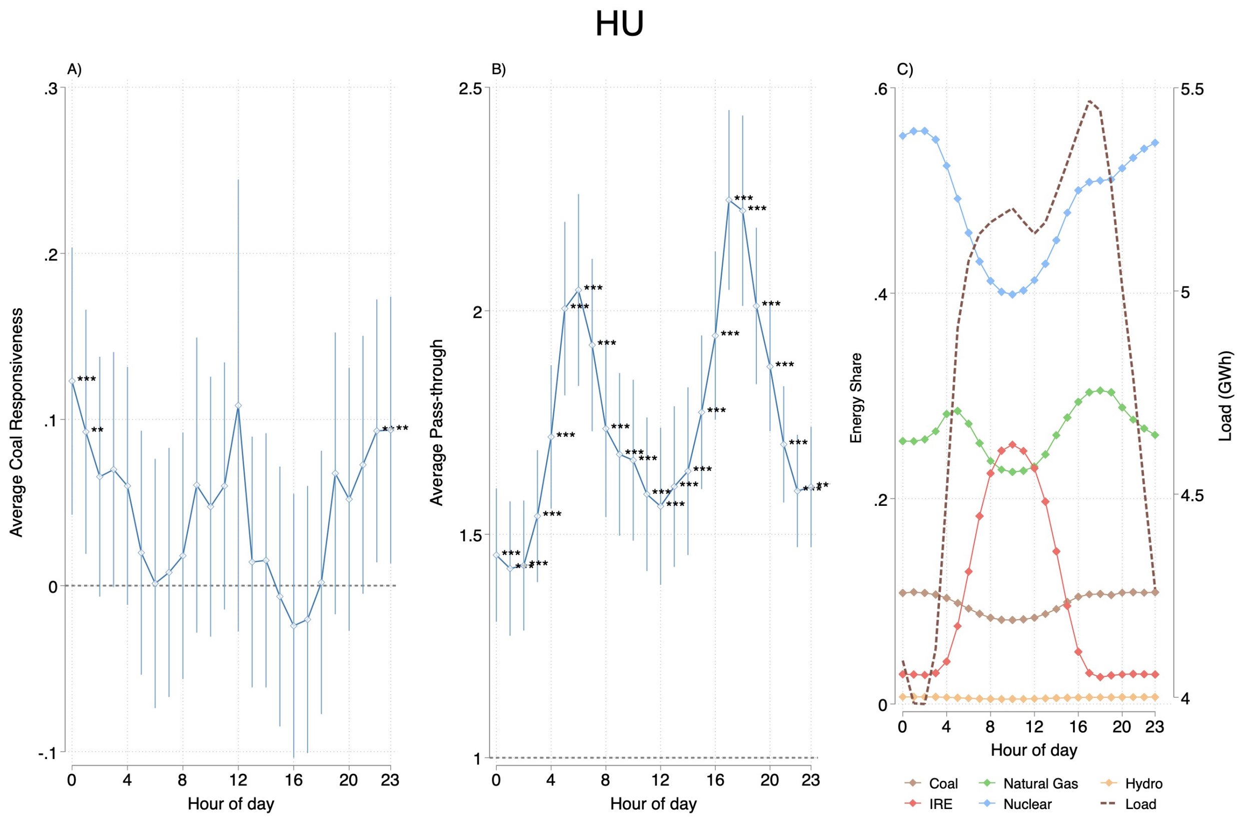

Supplementary Fig. 23: A) Estimated hourly coal responsiveness coefficients; B) estimated pass-through coefficients by hour; C) the average share of different generation technologies used by hour for Hungary, using our main specifications for each country (Supplementary Tables 28 and 29) while including an hourly interaction term with our regressor of interest. Dots in panels A) and B) represent the estimated hourly coal responsiveness or pass-through coefficients respectively, while the vertical lines capture 95% confidence intervals (± 1.96 times the standard error of each point estimate). The statistical test used is a two-sided t-test. The coefficients that are statistically distinct from either 0 or 1 at the 1% or 5% significance level are indicated with three stars or two stars, respectively. In the right panel, "intermittent renewable" includes solar, wind, and hydro-run-of-river. The dashed line displays the average hourly load.

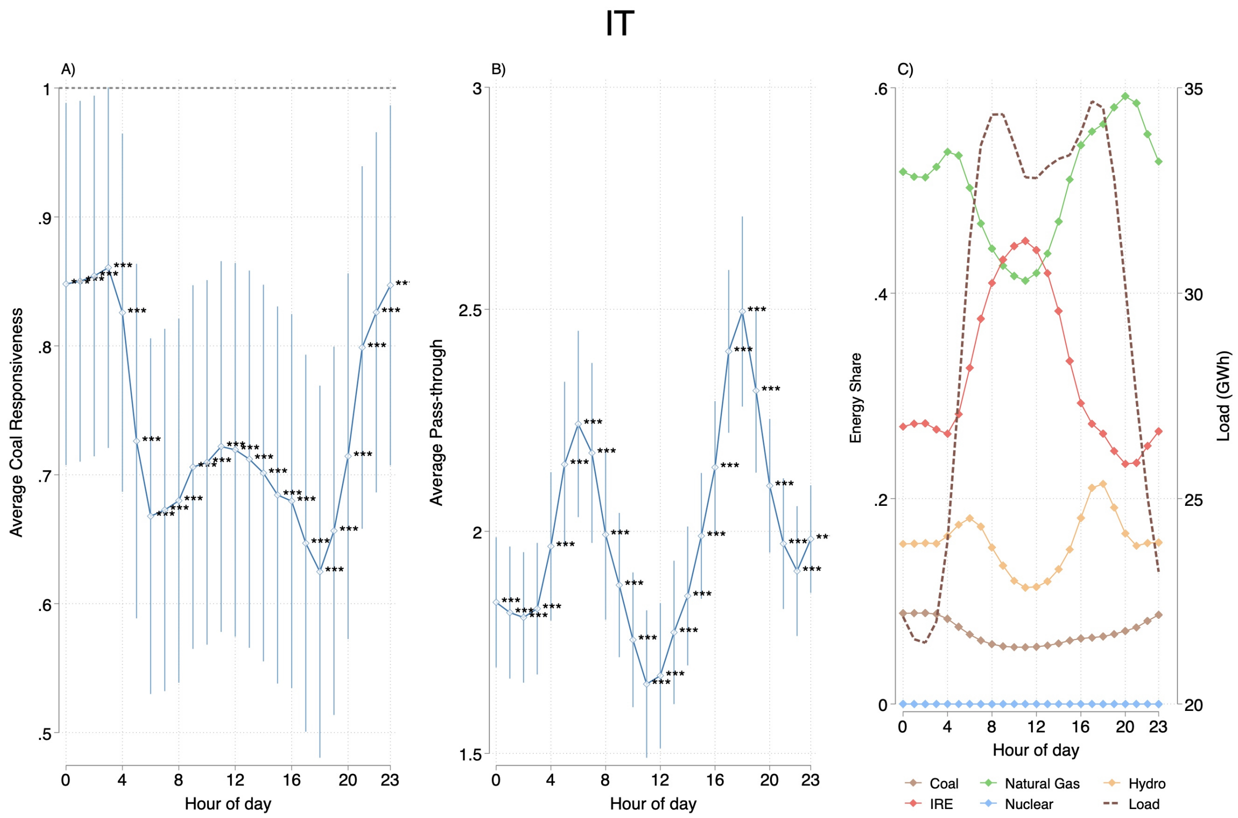

Supplementary Fig. 24: A) Estimated hourly coal responsiveness coefficients; B) estimated pass-through coefficients by hour; C) the average share of different generation technologies used by hour for Italy, using our main specifications for each country (Supplementary Tables 28 and 29) while including an hourly interaction term with our regressor of interest. Dots in panels A) and B) represent the estimated hourly coal responsiveness or pass-through coefficients respectively, while the vertical lines capture 95% confidence intervals (±1.96 times the standard error of each point estimate). The statistical test used is a two-sided t-test. The coefficients that are statistically distinct from either 0 or 1 at the 1% or 5% significance level are indicated with three stars or two stars, respectively. In the right panel, "intermittent renewable" includes solar, wind, and hydro-run-of-river. The dashed line displays the average hourly load.

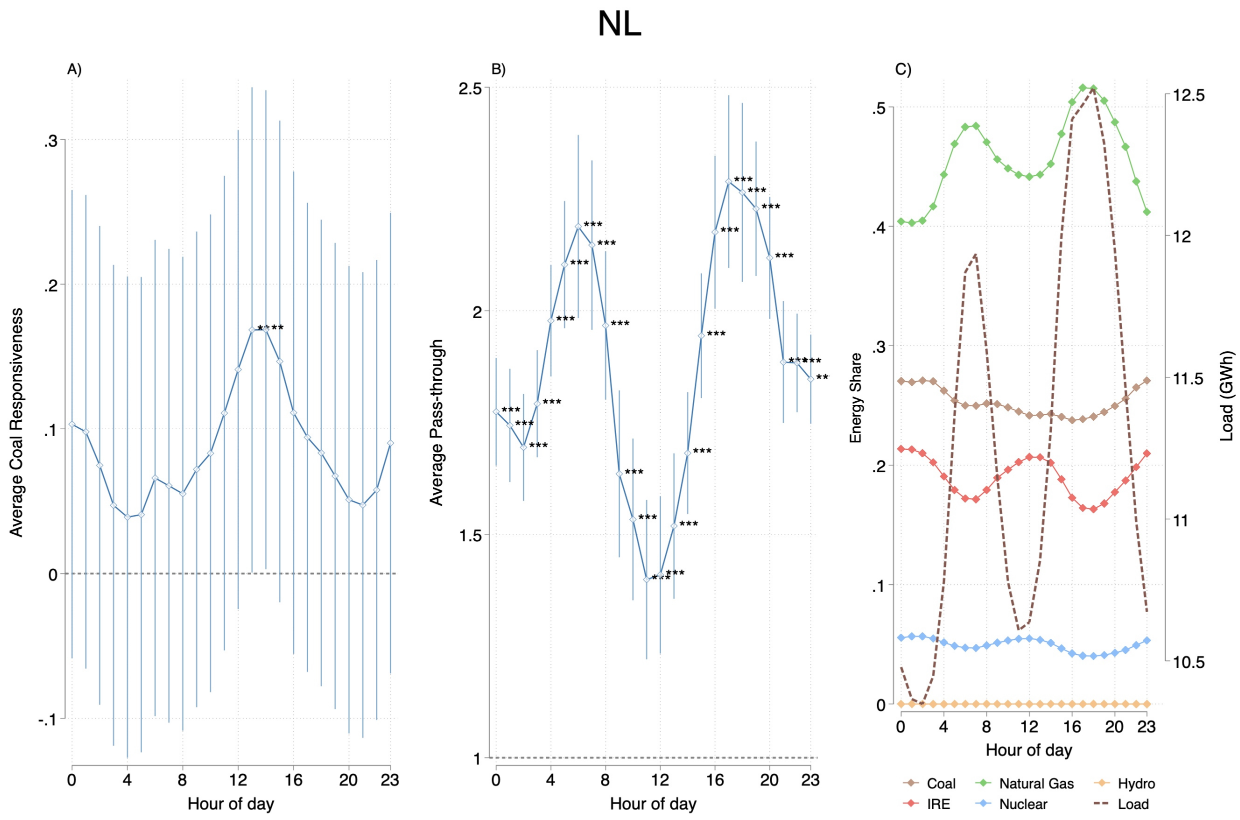

Supplementary Fig. 25: A) Estimated hourly coal responsiveness coefficients; B) estimated pass-through coefficients by hour; C) the average share of different generation technologies used by hour for the Netherlands, using our main specifications for each country (Supplementary Tables 28 and 29) while including an hourly interaction term with our regressor of interest. Dots in panels A) and B) represent the estimated hourly coal responsiveness or pass-through coefficients respectively, while the vertical lines capture 95% confidence intervals (±1.96 times the standard error of each point estimate). The statistical test used is a two-sided t-test. The coefficients that are statistically distinct from either 0 or 1 at the 1% or 5% significance level are indicated with three stars or two stars, respectively. In the right panel, “intermittent renewable” includes solar, wind, and hydro-run-of-river. The dashed line displays the average hourly load.

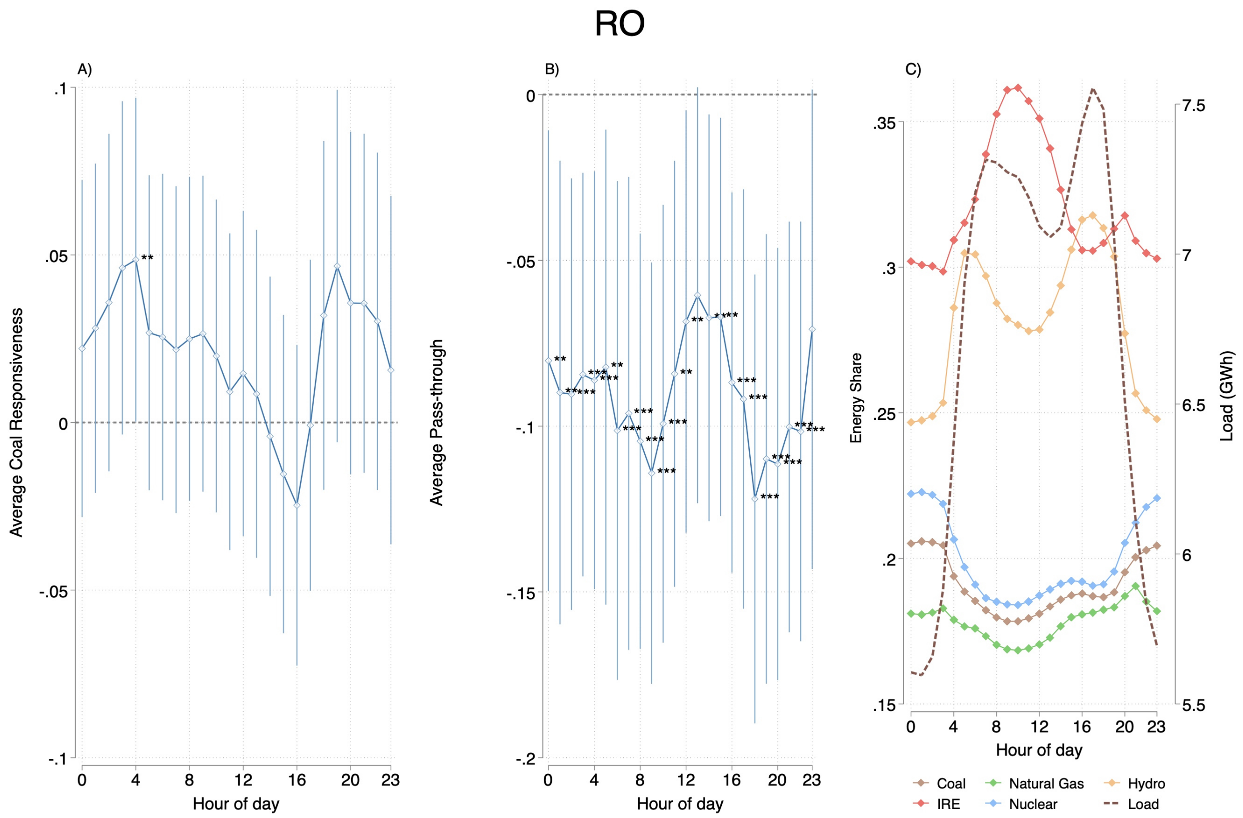

Supplementary Fig. 26: A) Estimated hourly coal responsiveness coefficients; B) estimated pass-through coefficients by hour; C) the average share of different generation technologies used by hour for Romania, using our main specifications for each country (Supplementary Tables 28 and 29) while including an hourly interaction term with our regressor of interest. Dots in panels A) and B) represent the estimated hourly coal responsiveness or pass-through coefficients respectively, while the vertical lines capture 95% confidence intervals (± 1.96 times the standard error of each point estimate). The statistical test used is a two-sided t-test. The coefficients that are statistically distinct from either 0 or 1 at the 1% or 5% significance level are indicated with three stars or two stars, respectively. In the right panel, "intermittent renewable" includes solar, wind, and hydro-run-of-river. The dashed line displays the average hourly load.

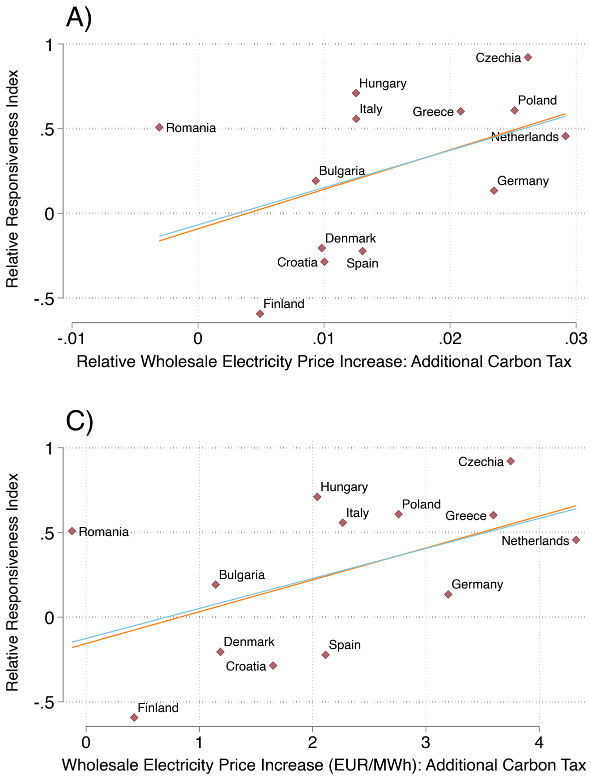

Supplementary Fig. 27: **A)** Relationship between the relative responsiveness index and the relative wholesale price change (compared to a pre-crisis baseline price) under an additional carbon tax (12.18 EUR/tonne), during 2022. **B)** Relationship between the relative responsiveness index and relative wholesale price change (compared to a pre-crisis baseline price) under 180 EUR/MWh Gas Cap, during 2022. **C)** Relationship between the relative responsiveness index and the wholesale price change under an additional carbon tax (12.18 EUR/tonne), during 2022. **D)** Relationship between the relative responsiveness index and wholesale price change under 180 EUR/MWh Gas Cap, during 2022.

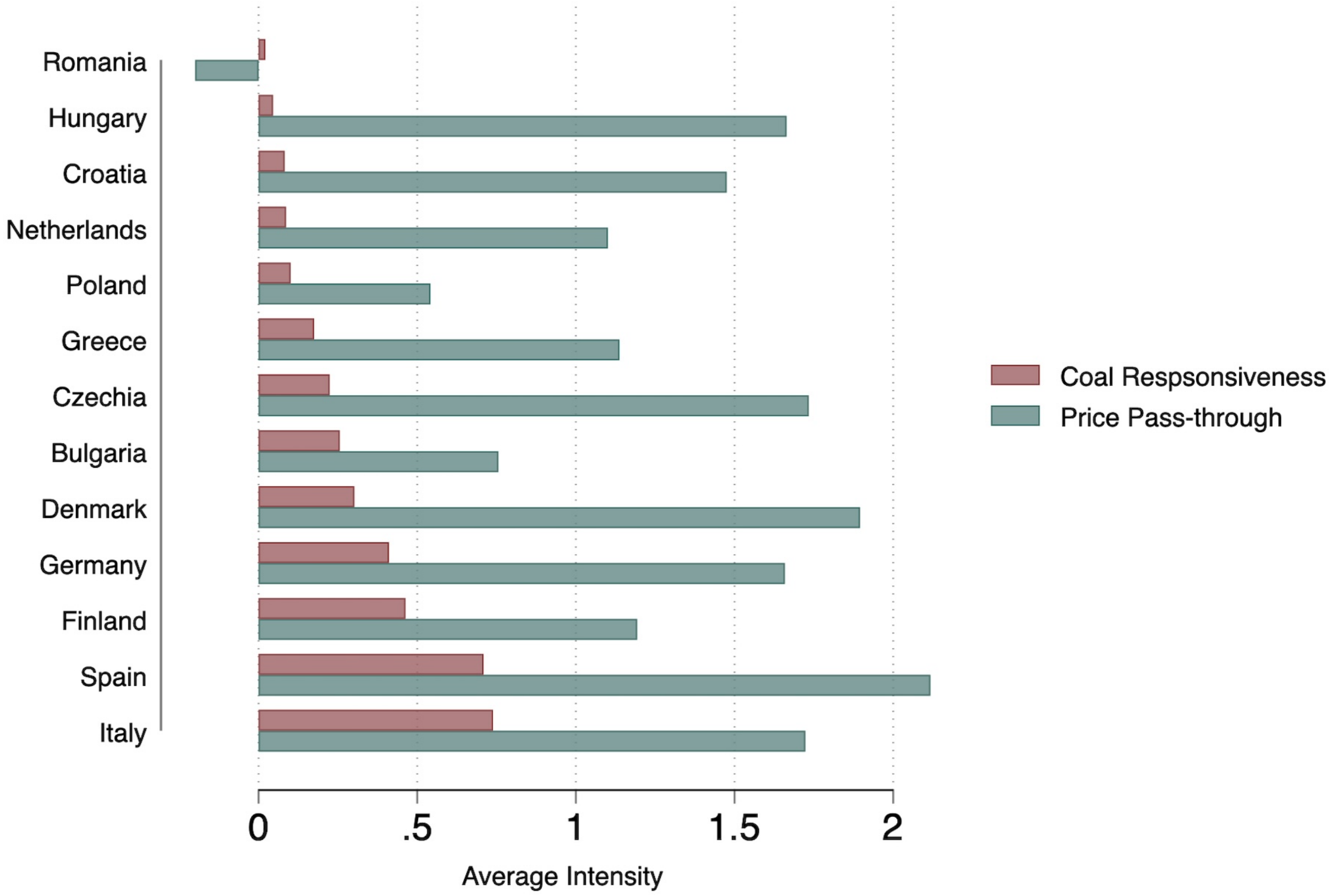

Supplementary Fig. 28: Average intensity (regression coefficients) of coal responsiveness and natural gas price pass-through for all countries during our sample period.

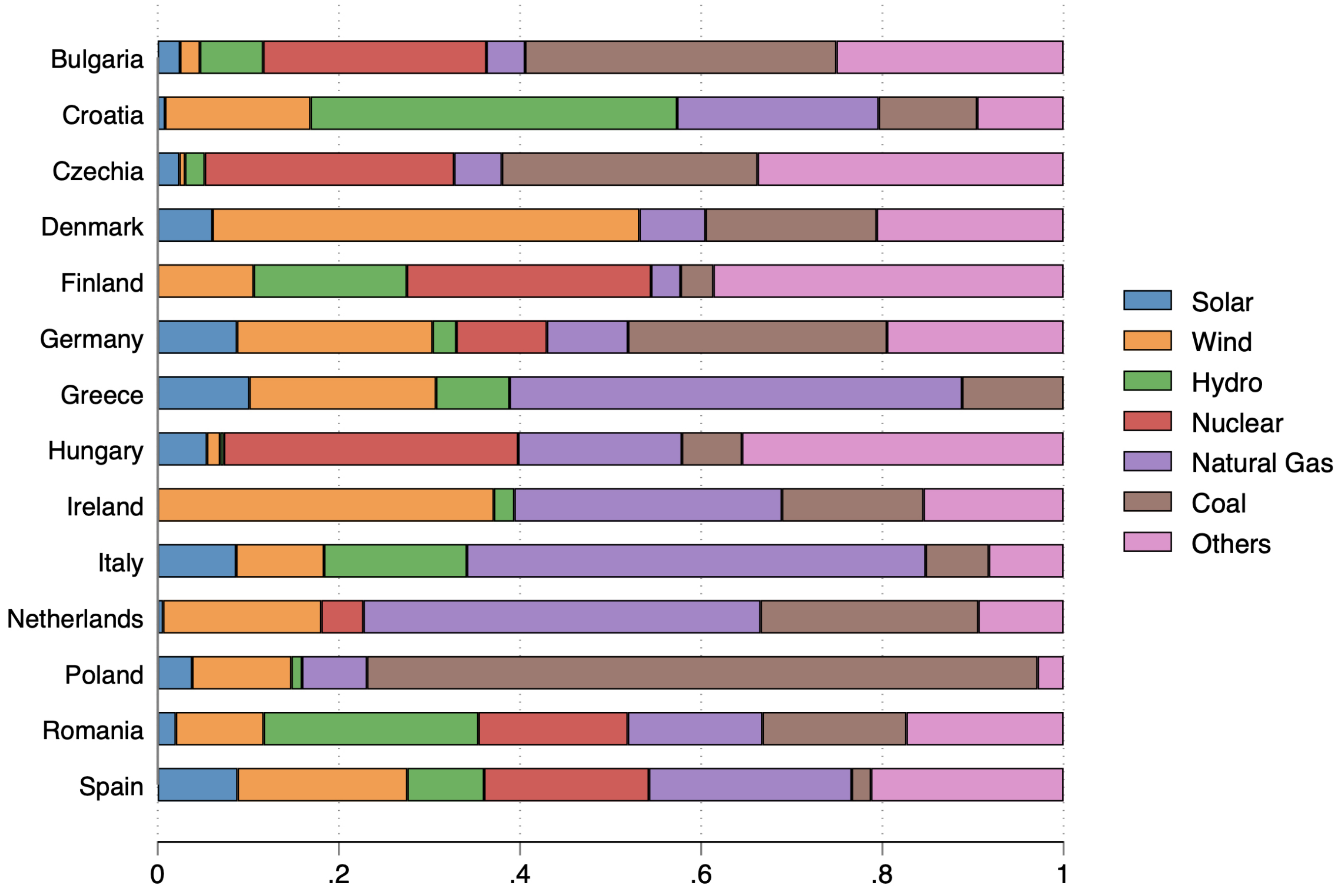

Supplementary Fig. 29: Illustration of the energy generation mix for all countries during our sample period. Coal refers to both hard coal and lignite, as used in our analysis, while Hydro refers to both dispatchable and run-of-river.

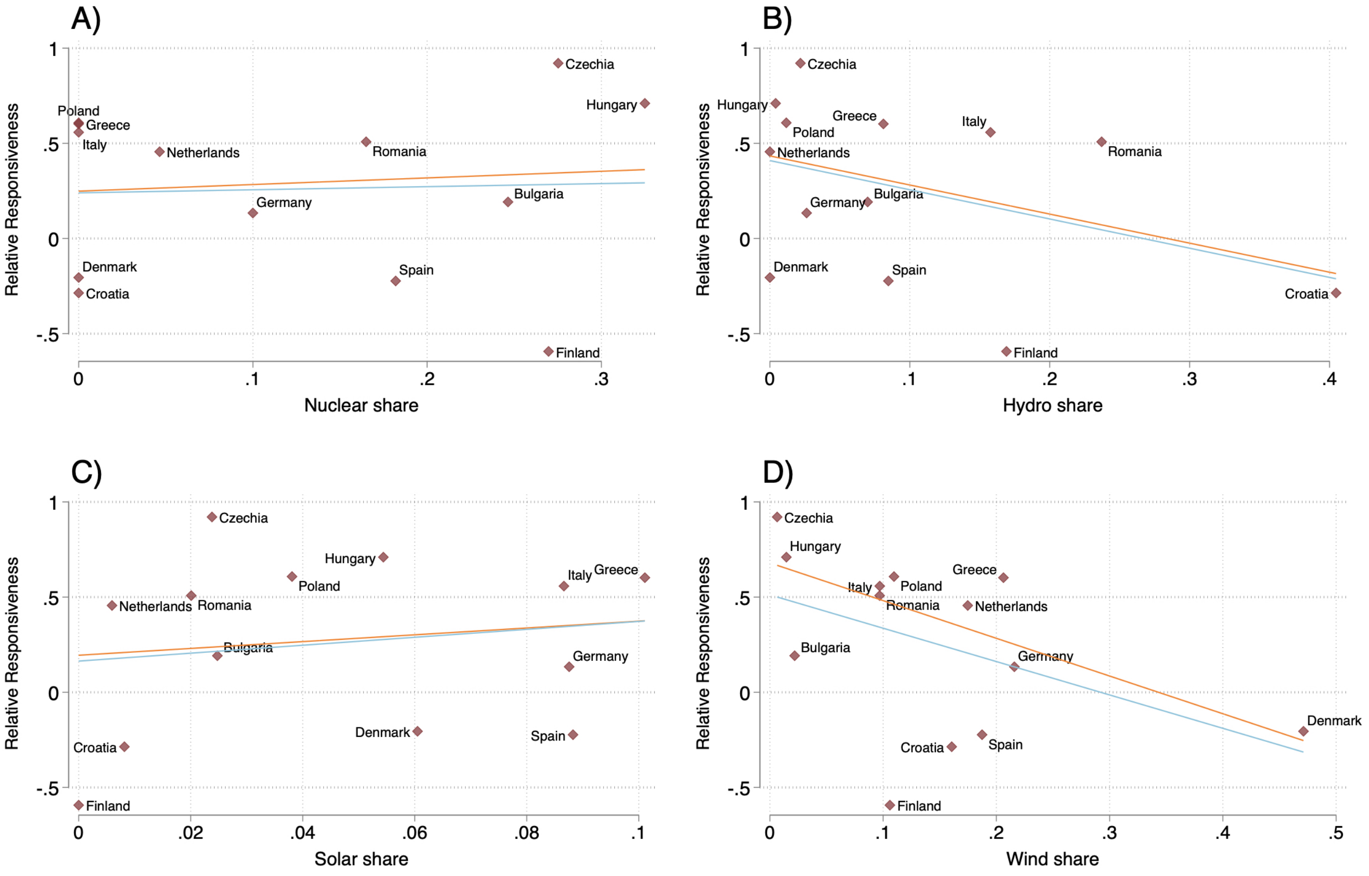

Supplementary Fig. 30: Relationship between relative responsiveness and breakdown of decarbonized energy sources. A) Relationship between the relative responsiveness index and the nuclear energy generation share. **B)** Relationship between the relative responsiveness index and the hydro energy generation share. **C)** Relationship between the relative responsiveness index and the solar energy generation share. **D)** Relationship between the relative responsiveness index and the wind energy generation share.

Supplementary References

[1] David C Broadstock, Raymond Li, and Linjin Wang. Integration reforms in the european natural gas market: a rolling-window spillover analysis. *Energy Economics*, 92:104939, 2020.

[2] Patrick Heather. *European traded gas hubs: German hubs about to merge*. Number 170. OIES Paper: NG, 2021.

[3] Patrick Heather. European traded gas hubs: the supremacy of ttf, 2020.

[4] A Neumann and A Cullmann. What's the story with natural gas markets in europe? empirical evidence from spot trade data. In *2012 9th International Conference on the European Energy Market*, pages 1–6. IEEE, 2012.

[5] Frank Asche, Bård Misund, and Marius Sikveland. The relationship between spot and contract gas prices in europe. *Energy Economics*, 38:212–217, 2013.

[6] Patrick Heather. The evolution of european traded gas hubs. 2016.

[7] Rafael Garaffa, Alexandre Szklo, André FP Lucena, and José Gustavo Féres. Price adjustments and transaction costs in the european natural gas market. *The Energy Journal*, 40(1), 2019.

[8] Vera Jotanovic and Rita Laura D'Ecclesia. The european gas market: new evidences. *Annals of Operations Research*, 299(1):963–999, 2021.

[9] Beatriz Martínez and Hipòlit Torró. Hedging spark spread risk with futures. *Energy Policy*, 113:731–746, 2018.

[10] Gary W Emery and Qingfeng Liu. An analysis of the relationship between electricity and natural-gas futures prices. *Journal of Futures Markets: Futures, Options, and Other Derivative Products*, 22(2):95–122, 2002.

[11] Petr Spodniak and Valentin Bertsch. Is flexible and dispatchable generation capacity rewarded in electricity futures markets? a multinational impact analysis. *Energy*, 196:117050, 2020.

[12] Param Silvapulle and Imad A Moosa. The relationship between spot and futures prices: evidence from the crude oil market. *Journal of Futures Markets: Futures, Options, and Other Derivative Products*, 19(2):175–193, 1999.

[13] Martin T Bohl, Christian A Salm, and Michael Schuppli. Price discovery and investor structure in stock index futures. *Journal of Futures Markets*, 31(3):282–306, 2011.

[14] Sebastian Nick. The informational efficiency of european natural gas hubs: Price formation and intertemporal arbitrage. *The Energy Journal*, 37(2), 2016.

[15] Yevheniia Sribna, Olena Trokhymets, Ihor Nosatov, and Iryna Kriukova. The globalization of the world coal market–contradictions and trends. In *E3S Web of Conferences*, volume 123, page 01044. EDP Sciences, 2019.

[16] Martha Roggenkamp and François Boisseleau. The liberalisation of the eu electricity market and the role of power exchanges. *Regulation*, 4:2, 2005.

[17] Jean-Michel Glachant and Yannick Perez. The liberalization of electricity markets. In *International Handbook of Network Industries*. Edward Elgar Publishing, 2011.

[18] EU4Energy Initiative. Electricity market functions – short overview and description. Technical report, March 22, 2022.

[19] Raul Bajo-Buenestado, Antonio M. Bento, Daniel Kaffine, and Zissis E. Marmarelis. Decarbonization and electricity price vulnerability. *Nature Sustainability*, 2025.

[20] Daniel T Kaffine and Graham A Davis. A multi-row deletion diagnostic for influential observations in small-sample regressions. *Computational Statistics & Data Analysis*, 108:133–145, 2017.
